# Development and validation of a machine learning risk prediction model for asthma attacks in adults in primary care

**DOI:** 10.1038/s41533-025-00428-8

**Published:** 2025-04-23

**Authors:** Holly Tibble, Aziz Sheikh, Athanasios Tsanas

**Affiliations:** 1 Usher Institute, The University of Edinburgh, Edinburgh, UK; 2 Asthma UK Centre for Applied Research, Edinburgh, UK

**Keywords:** Epidemiology, Prognosis, Asthma, Epidemiology

## Abstract

Primary care consultations provide an opportunity for patients and clinicians to assess asthma attack risk. Using a data-driven risk prediction tool with routinely collected health records may be an efficient way to aid promotion of effective self-management, and support clinical decision making. Longitudinal Scottish primary care data for 21,250 asthma patients were used to predict the risk of asthma attacks in the following year. A selection of machine learning algorithms (i.e., Naïve Bayes Classifier, Logistic Regression, Random Forests, and Extreme Gradient Boosting), hyperparameters, training data enrichment methods were explored, and validated in a random unseen data partition. Our final Logistic Regression model achieved the best performance when no training data enrichment was applied. Around 1 in 3 (36.2%) predicted high-risk patients had an attack within one year of consultation, compared to approximately 1 in 16 in the predicted low-risk group (6.7%). The model was well calibrated, with a calibration slope of 1.02 and an intercept of 0.004, and the Area under the Curve was 0.75. This model has the potential to increase the efficiency of routine asthma care by creating new personalized care pathways mapped to predicted risk of asthma attacks, such as priority ranking patients for scheduled consultations and interventions. Furthermore, it could be used to educate patients about their individual risk and risk factors, and promote healthier lifestyle changes, use of self-management plans, and early emergency care seeking following rapid symptom deterioration.

## Introduction

Asthma attacks are the cause of more than 25 deaths per week on average in the UK^1^. If a patient contacts a health professional promptly following a decline in symptoms, short courses of systemic steroids can be prescribed on top of preventative therapy to relieve exacerbations and reduce the need for (transfer to or continuation in) emergency care^2,3^. Most asthma attacks occur in those who would be classed as having mild-to-moderate asthma^4^, due to the volume of such patients: only 5–10% of those with asthma are typically classed as having severe asthma^5^. Many patients in danger of life-threatening deterioration are unsure of how to handle emergency situations^6^, as highlighted by the finding in the 2014 UK National Review of Asthma Deaths which reported that only 55% of people who died from asthma had called for or received any medical assistance after their attack began^7^.

Consultations with GPs and asthma nurses in primary care present a key opportunity for the joint evaluation of asthma attack risk, and outline plans for action in the event of symptom deterioration^8^, as recommended by national asthma guidelines^9,10^. A personalised health promotion tool may be able to promote risk-reducing lifestyle choices, instigate revisions to asthma action plans, improve patient engagement with self-management protocols, and reduce patient anxiety^11–13^. Personalised guidance has been demonstrated to have a greater impact on disease prevention than generic documents^8^.

Machine learning algorithms can identify complex patterns in data from patient’s histories and symptoms that are associated with increased risk of severe outcomes. They often require high volumes of data to make such inferences without guidance from a clinical expert, which makes their application to the wealth of information recorded in routinely collected Electronic Health Records (EHRs) a promising pathway for research^14,15^. However, even in large national datasets, the infrequency of asthma attacks relative to the prevalence of asthma means that it can be challenging to identify potentially causal relationships that may lead to increased probability of an asthma attack. Furthermore, the imbalance between positive (attack) and negative (no attack) samples in the data poses a practical challenging setting for developing predictive models^16–18^. Therefore, many prediction models report either low (such as below 50%) *sensitivity* (predicted risk in people who had asthma attacks) or *positive predictive value* (incidence of asthma attacks in people with high predicted risk)^19^.

In this paper, we utilised the wealth of data recorded in EHRs and tested multiple methodologies for overcoming this data imbalance problem, to develop, validate and test a model for predicting whether asthma attacks would occur within the next year from a primary care-based asthma-related consultation.

## Methods

### Data

**Fig. 1:**
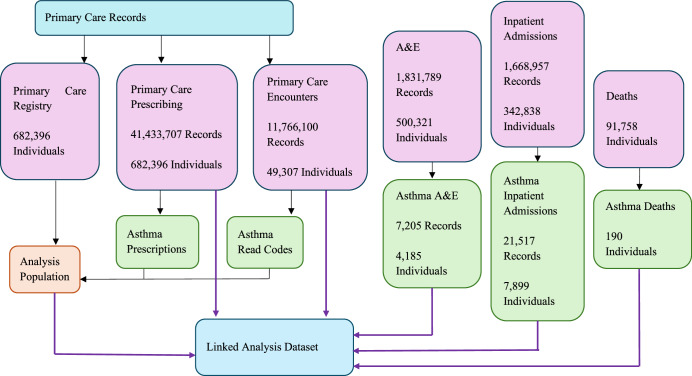
Linked Analysis Dataset Flow Diagram.

The Asthma Learning Healthcare System (ALHS) study recruited over half a million patients from 75 general practices in Scotland, with primary care records linked to national accident and emergency (A&E), hospital, and mortality datasets^20^. The original study period was between January 2009 and March 2017. The initial data processing report is provided in Supplementary Material A. The linked analysis dataset flow diagram is presented in Fig. 1.

### Analysis population

The analysis population for this study was adults (aged 18 and over), diagnosed with asthma in primary care, and treated with inhaled corticosteroids (ICS)^21^. The exclusion criteria were having missing age or sex in the primary care registration record.

Eachsample in the final analysis dataset was a day on which a primary care consultation related to asthma or a respiratory infection occurred, without an oral corticosteroid (OCS) prescription or secondary care asthma encounter. Therefore, we additionally excluded individuals who had no such event during their follow-up (as defined in Supplementary Material A).

Finally, those with a diagnosis of Chronic Obstructive Pulmonary Disease (COPD) were identified, and the time between first asthma diagnosis and first COPD diagnosis was estimated. A diagnosis of COPD prior to a diagnosis of asthma excluded patients from primary analyses, however they were retained for a sensitivity analysis (model testing only, no data included in model training). Similarly, for those with a diagnosis of COPD following their asthma diagnosis, the time (and any samples) after their COPD diagnosis was excluded from model training, but was retained for sensitivity analysis (model testing only).

### Outcome ascertainment

The model’s outcome was asthma attacks occurring within one year from the index date of each samples. The joint American Thoracic Society (ATS) and European Respiratory Society (ERS) Task Force definition of a severe exacerbation^22^ was used to define an asthma attack: a prescription of OCS, an asthma-related A&E visit, or an asthma-related hospital admission (ICD-10 codes J45 and J46). In addition, deaths with asthma as the primary cause were considered indicative of an asthma attack. The identification of asthma-related A&E presentations, inpatient admissions, and deaths is described in Supplementary Material A.

Prescriptions for OCS were considered indicative of an asthma attack if all of the following conditions were also met: 1) they were prescribed to someone with a diagnosis of asthma or receiving asthma treatment, 2) they were prescribed on the same day as an asthma-related consultation, 3) the prescribed strength was greater than or equal to 5 mg per dose, and 4) the total prescribed dose was between 50 and 350 mg.

### Prediction model features

Supplementary Material B describes the full set of risk factors that were included in the analysis, and notes regarding the feature extraction method and missing data handling.

### Training data enrichment

As described in the introduction, the low incidence of asthma attacks in the general asthma population results in complexity in model development which can often result in poor model sensitivity. Herein, we have tested the utility of the training data enrichment method known as SMOTEing^23,24^, with three difference parameter sets as described in Supplementary Material C.

### Analysis plan

**Fig. 2:**
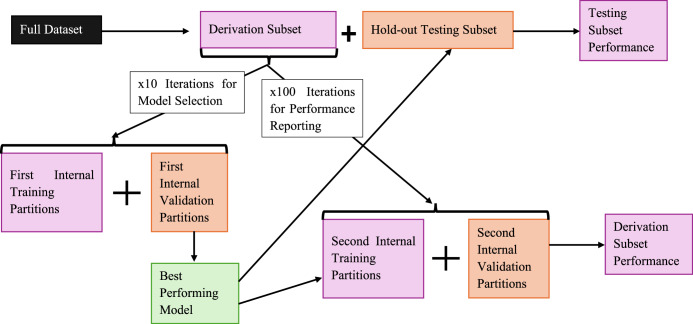
Diagram of Dataset Partitioning for Model Training, Model Selection, Internal Validation Performance Reporting, and Testing Partition Performance Reporting.

In this analysis, a random partition approach was used to split the population for model training and testing, as shown in Fig. 2. A random 90% partition of the ALHS dataset population was used for hyper-parameter optimisation, model selection and initial performance reporting (henceforth the *derivation subset*, n = 523,611 samples), and the remaining 10% was held-out for assessing model generalisation (*hold-out testing subset*, n = 63,331 samples). During the model selection process, the derivation dataset was randomly partitioned another 100 times, again with 90% of the data used for training the final selected algorithm (approximately 471,000 samples), hyper-parameters, and enrichment method, and 10% for internal validation (approximately 52,000 samples).

Four algorithms were tested, namely Naïve Bayes Classifier, Logistic Regression, Random Forests, and Extreme Gradient Boosting; these included variations based on training data enrichment approaches, algorithm hyper-parameters, and classification thresholds. The full model selection process is detailed in Supplementary Material C.

For each iteration, model, and enrichment method, the Area Under the Receiver-Operator Curve (AUC) was calculated. The confusion matrix was also recorded, based on the primary classification threshold that optimised the Matthews Correlation Coefficient (MCC; identified using golden-section search optimisation^25^) in predictions made on the training data partition. The MCC was used as the primary performance measure as it utilises the balance ratios of all four categories of the confusion matrix (i.e., true positives, true negatives, false positives, and false negatives). The other performance measures reported were: sensitivity, specificity, positive predictive value (PPV), negative predictive value (NPV), accuracy, and balanced accuracy. Across iterations, summary statistics were calculated for each performance measure to provide some estimate of the average performance, and the certainty around that estimate.

As a sensitivity analysis of the classification threshold selection, a further three approaches were explored: 1) the default (probability greater than 0.5) classification threshold (aka ‘Fixed’), 2) the classification threshold closest to the outcome prevalence in the training data partition, to 3 decimal places (aka ‘Prevalence’), and 3) the mean of the ‘Variable’ and ‘Prevalence’ values (aka ‘Balanced’).

The model performance was summarised over the 100 iterations of the split-sample process. Calibration was assessed using the slope and intercept of a logistic regression model between the predicted risk and the observed outcome. Model coefficients were calculated across the models trained from the 100 iterations of derivation data partitioning.

Finally, the model was then retrained on the full derivation dataset and tested on the as-yet unseen holdout partition. The performance was also reported with data stratified by various risk factors. These were: (i) history of other comorbid chronic pulmonary disease, (ii) BTS Step (to evaluate the decision to assign the level ‘0’ to periods of non-adherence), (iii) missingness of peak flow and blood eosinophil measurements (to evaluate their added value), (iv) smoking status (to evaluate the utility of assigning the level ‘never’ having smoked to those with missing smoking status), (v) recent respiratory infections, oral steroid prescriptions and prior known asthma attacks (to establish the utility of the model in predicting those not known to be prone to attacks), and (vi) if or when in the future the patient was diagnosed with COPD. In the latter case, this information obviously could not be known at the time of prediction, however we explored it to examine the potential impact of over-lapping diagnosis, which might be considered in individuals at high risk of COPD.

### Reporting

Deviations from the protocol paper, published in BMJ Open in 2019^26^, are listed in Supplementary Material D.

This work was written in line with guidance from RiGoR (Reporting Guidelines to address common sources of bias in Risk model development, by Kerr et al.^27^), TRIPOD (Transparent Reporting of a multivariable prediction model for Individual Prognosis Or Diagnosis, by Collins et al.^28^), and RECORD (Reporting of studies Conducted using Observational Routinely-collected health Data, by Benchimol et al.^29^). Checklists are presented in Supplementary Material E.

## Results

### Analysis population

**Fig. 3:**
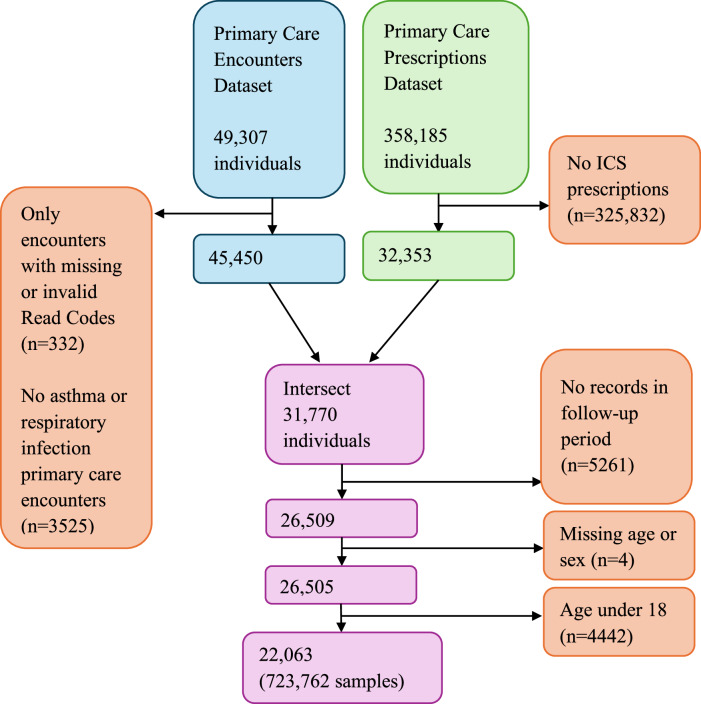
Asthma attack risk prediction model analysis population flow diagram.

There were 22,063 patients included in the study, with 723,762 samples, as illustrated in Fig. 3. There were 19,125 individuals in the training data partition, with a total of 584,288 samples (eligible consultations) spanning 115,282.5 person-years (median 7.0 years per person, interquartile range (IQR) 5.1 to 7.4, and range <0.1 to 7.5 years). 19,283 samples were excluded as they occurred after a diagnosis of COPD had been made.

There were 2125 individuals in the testing data partition, with a total of 65,985 samples spanning 12,919.0 person-years (median 7.0 years per person, interquartile range 5.2 to 7.4, and range 0.1 to 7.6 years).

Finally, there were 871 individuals in the post-COPD diagnosis testing data partition (sensitivity analyses). 813 of these were individuals who had no samples prior to their subsequent COPD diagnosis, and samples post COPD diagnosis from 58 individuals in the testing data partition were also included. There were a total of 54,206 samples spanning 5030.0 person-years (median 7.0 years per person, interquartile range 4.3 to 7.4, and range 0.3 to 7.6 years). The demographics of each data partition at baseline are presented in Table 1. Supplementary Material F shows the demographics (and all other characteristics) by sample in each partition.

**Table 1:** Demographics of the ALHS analysis population.

Characteristics	Training Data Patient Sample (N = 19,125)	Testing Data Patient Sample (N = 2125)	COPD-Overlap Patient Sample (N = 871)
Prevalence of Asthma Attacks within One Year of Index Date			
	938 (4.90%)	94 (4.42%)	83 (9.53%)
Prevalence of 1+ Asthma Attacks within Study Follow-up			
	3311 (17.31%)	366 (17.22%)	304 (34.90%)
Baseline Age^a^			
18 to 35	6264 (32.75%)	718 (33.79%)	4 (0.46%)
36 to 45	3697 (19.33%)	407 (19.15%)	53 (6.08%)
46 to 60	5012 (26.21%)	552 (25.98%)	238 (27.32%)
61 to 75	3109 (16.26%)	345 (16.24%)	394 (45.24%)
76 to 99	1043 (5.45%)	103 (4.85%)	182 (20.90%)
Sex			
Male	7624 (39.86%)	850 (40.00%)	364 (41.79%)
Female	11501 (60.14%)	1275 (60.00%)	507 (58.21%)
Baseline Scottish Index of Multiple Deprivation			
1 (Highest Deprivation)	4134 (21.62%)	476 (22.4%)	257 (29.51%)
2	3763 (19.68%)	433 (20.38%)	213 (24.45%)
3	3154 (16.49%)	345 (16.24%)	137 (15.73%)
4	4260 (22.27%)	454 (21.36%)	156 (17.91%)
5 (Lowest Deprivation)	3356 (17.55%)	371 (17.46%)	90 (10.33%)
Missing	458 (2.39%)	46 (2.16%)	18 (2.07%)
Baseline Scottish Urban Rural Classification			
1 (Large Urban)	6396 (33.44%)	711 (33.46%)	255 (29.28%)
2 (Other Urban Area)	6747 (35.28%)	749 (35.25%)	340 (39.04%)
3 (Accessible Small Towns)	1596 (8.35%)	202 (9.51%)	111 (12.74%)
4 (Remote Small Towns)	689 (3.60%)	72 (3.39%)	21 (2.41%)
5 (Accessible Rural)	2026 (10.59%)	211 (9.93%)	81 (9.30%)
6 (Remote Rural)	1107 (5.79%)	122 (5.74%)	42 (4.82%)
Missing	564 (2.95%)	58 (2.73%)	21 (2.41%)
Baseline BTS Treatment Step			
0 (No controllers)	7232 (37.81%)	780 (36.71%)	330 (37.89%)
1	4354 (22.77%)	482 (22.68%)	23 (2.64%)
2	1164 (6.09%)	156 (7.34%)	50 (5.74%)
3	3683 (19.26%)	416 (19.58%)	124 (14.24%)
4	2692 (14.08%)	291 (13.69%)	344 (39.49%)
Baseline Comorbidities^b^			
Rhinitis	61 (0.32%)	4 (0.19%)	1 (0.11%)
Nasal Polyps	7 (0.04%)	0	1 (0.11%)
GERD^c^	19 (0.1%)	1 (0.05%)	3 (0.34%)
Eczema	83 (0.43%)	9 (0.42%)	3 (0.34%)
Chronic pulmonary disease	14 (0.07%)	1 (0.05%)	7 (0.8%)
Anxiety/Depression	213 (1.11%)	30 (1.41%)	12 (1.38%)
Obesity	567 (2.96%)	64 (3.01%)	55 (6.31%)

^a^Age categorisation is presented in this table for ease of viewing, however continuous values are used in the model.

^b^Diagnoses of eczema, rhinitis, nasal polyps, and anxiety and/or depression in the last five years were included in this table.

^c^Diagnoses of Gastro-Esophageal Reflux Disease (GERD) in the last year was included in this table.

There was a median of 34 days between subsequent consultations in the training data partition (IQR 18 to 60 days), and the incidence of asthma attacks in the year after consultation was 8.0% (n = 46921/584288).

### Model testing

The results of the model selection process are reported in Supplementary Material G. The final selected model used the logistic regression algorithm, with no training data enrichment, and the ‘balanced’ threshold for classification.

In Table 2, the summary statistics of a selection of model performance measures across the 100 iterations of derivation data partitioning are presented.

**Table 2:** Summary statistics of model performance measures from 100 internal validation data partitions, and the hold-out data partition.

Performance Measure	ALHS Model Development Data					ALHS Hold-out Validation Data
	Minimum	Lower Quartile	Median	Upper Quartile	Maximum	
Sensitivity	20.7	26.2	28.9	30.5	36.5	30.1
Specificity	93.9	94.6	94.9	95.2	96.0	94.9
PPV	26.1	30.4	32.4	34.7	44.2	36.2
NPV	92.3	93.6	93.9	94.2	94.6	93.3
Accuracy	87.5	89.3	89.6	90.0	90.9	89.1
AUC	71.6	73.8	74.9	75.9	78.4	75.0
Balanced Accuracy	57.9	60.6	61.9	62.6	66.2	62.5
MCC	18.0	22.6	25.2	26.6	35.3	27.1
Outcome Prevalence	7.8	8.0	8.0	8.1	8.2	8.9

The final threshold used for classification in the holdout partition was 0.199: the median across the 100 derivation partition iterations (range 0.133–0.234, interquartile range = 0.174 – 0.216). As shown in the final column of Table 2, the performance in the holdout partition, following a full retrain of the model on the entire derivation dataset, was consistent with the range observed in the derivation dataset. This internal validation demonstrated the stability of the model performance within these data to perturbations of the sample set and confirmed that the crossover in samples between the model selection and performance reporting subsets did not bias the results.

**Fig. 4:**
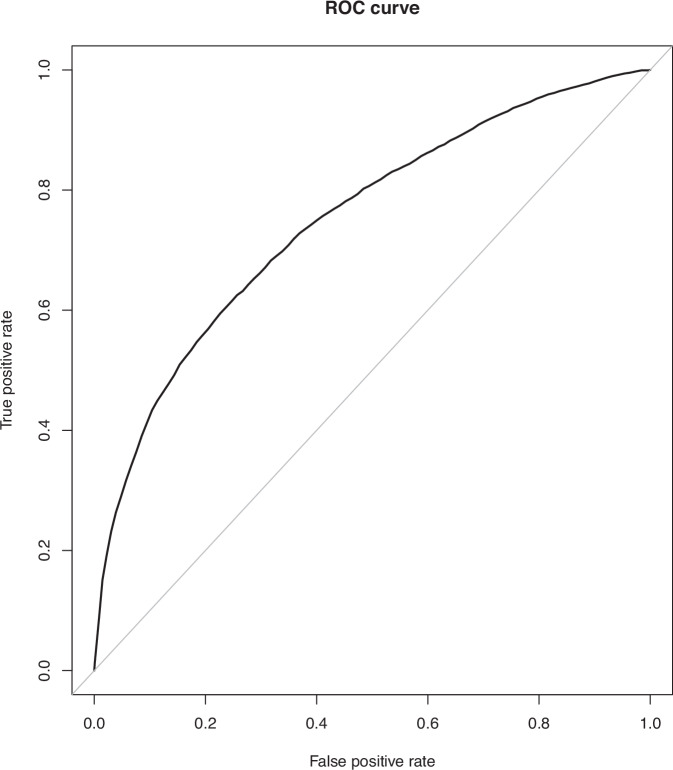
Receiver-Operator Curve.

Around 1 in 3 (PPV = 36.2%) predicted high-risk patients had an attack within one year of consultation, compared to approximately 1 in 16 in the predicted low-risk group (6.7%). The Receiver-Operator Curve (ROC) is presented in Fig. 4. The confusion matrix in the holdout partition is presented in Table 3. The model had good calibration overall, with logistic regression between the predicted risk and observed outcome yielding a calibration intercept of 0.004 and slope of 1.02.

**Table 3:** Confusion matrix for model performance in holdout partition.

		Observed Outcome	
		Asthma Attack	No Asthma Attack
Predicted Class	High Risk	1758	4084
	Low Risk	3097	57,046

### Model coefficients

In the final model, history of asthma attacks recorded in primary care were strongly associated with risk of attacks in the next year: having an attack between 1 and 2 years before the index date resulted in 1.959 times higher odds of a future attack than no record of an attack in the last two years, and more recent historical attacks increased the odds further (Supplementary Material H). A recent history of respiratory infections was also associated with higher odds of attacks, with an infection 1 to 2 years prior to index date associated with an odds ratio of 1.494 compared to none in the past 2 years (and increasing odds with more recency). Results were highly consistent between the models trained in iterations of internal testing and the final model tested on the holdout partition.

The odds ratios for several comorbidities in the time window of ‘longer than 5 years ago’ were significantly lower than the reference categories of ‘never recorded’, which may be indicative of an artefact. As a sensitivity analysis, these features were recoded to ‘longer than 5 years ago or never recorded’. For anxiety/depression there was a trend that more recent records were associated with higher risk, however the results were not consistent in other comorbidities (data not shown). Further investigation may be warranted.

### Discrimination and calibration in population subgroups

Sensitivity was markedly higher in those with a history of comorbid chronic pulmonary disease (excluding asthma and COPD) than those without: 85.9% versus 29.4% (Table 4). Sensitivity was very high in those with multiple respiratory infections in either the current or previous calendar year (80.8%), those with multiple OCS prescriptions in either the current or previous calendar year (100%), and those with an asthma attack recorded in last 2 years (70.3%). However, the specificity for each of these subgroups was also lower: 52.2, 6.1, and 61.4%, respectively. The calibration in most of the population subgroups was good (plots presented in Supplementary Material I), except for those with recent oral steroid treatment or another concurrent chronic pulmonary disease.

**Table 4:** Subgroup Analyses.

Characteristic	Characteristic Value	Characteristic Prevalence (%)	Attack Rate in Samples	Sensitivity	Specificity	PPV	NPV
History of comorbid Chronic Pulmonary Disease	Yes	0.8	39.3	85.9	67.6	28.1	97.0
	No	99.2	7.1	29.4	95.1	36.6	93.3
Future COPD Diagnosis	None Recorded*	95.9	6.9	30.4	95.2	36.4	93.8
	Within 5 Years	2.0	14.3	36.6	90.1	42.2	87.8
	In more than 5 Years Time	2.1	21.6	22.8	78.8	29.2	72.7
More than one respiratory infection in previous calendar year or current calendar year to date	Yes	2.6	57.6	80.8	52.2	41.5	86.6
	No	97.4	60	25.2	95.7	34.8	93.4
BTS/SIGN Treatment Step	0	6.8	2.9	21.3	98.6	56.5	93.7
	1	21.7	1.3	8.2	99.0	28.3	95.9
	2	9.7	3.3	10.7	97.1	16.7	95.2
	3	27.6	5.2	16.9	95.8	25.1	93.3
	4	34.2	15	43.3	89.6	40.2	90.7
More than one asthma related consultation in previous calendar year, or current calendar year to date	Yes	13.1	29.8	60.9	78.3	42.1	88.5
	No	86.9	4.0	16.7	97.0	29.6	93.8
More than one OCS prescription in previous calendar year, or current calendar year to date	Yes	2.3	96.8	100	6.1	49.0	100
	No	97.7	5.2	20.1	96.1	30.6	93.3
Asthma attack recorded in primary care in the last 2 years	Yes	14.3	46.3	70.3	61.4	37.0	86.5
	No	85.7	0.8	3.8	99.4	28.7	93.9
Peak Flow Measurement in the last week	Yes	2.4	9.8	40.4	93.0	35.3	94.3
	No	97.6	7.3	29.8	94.9	36.2	93.3
Blood Eosinophil Measurement ever recorded	Yes	27.3	11.9	38.3	91.3	34.4	92.5
	No	72.7	5.7	26.1	96.2	37.7	93.6
Smoking Status	Current	14.9	12.9	29.9	89.5	28.2	90.3
	Former	16.7	12.1	42.8	91.7	39.0	92.8
	Never	68.4	5.0	25.7	96.7	39.1	94.0

*Those with no future COPD diagnosis recorded will have at least one year of follow-up after the observation date, but no further stratification by follow-up was conducted.

The model had lower sensitivity and PPV in those diagnosed with COPD more than 5 years from their observation date (22.8%), compared to those with no COPD recorded, or those with COPD recorded within 5 years of the observation date (30.4 and 36.6%, respectively). Those with COPD diagnosed more than five years after the observation date had poor calibration (Supplementary Material I).

## Discussion

### Summary of results

Our selected model used the logistic regression algorithm with no training data enrichment, and had an AUC of 0.75. The specificity and negative predictive value were high (95% and 93%), highlighting the model’s strength at accurately identifying a large proportion of patients at low risk. Around 1 in 3 predicted high-risk patients had an attack within one year of consultation (PPV = 36.2%), compared to approximately 1 in 16 in the predicted low-risk group, and there was good calibration (slope of 1.02). The model’s sensitivity was poor (30.1%) however, so most asthma attacks were not predicted in advance. We demonstrated the effect of various classification thresholds to dictate the balance between minimising false negatives (missed opportunities for intervention) and false positives (inefficient resource allocation).

### Results in context

Comparing model performance across studies is not straightforward due to differences in outcome definitions, populations, and even simply performance reporting, as highlighted in our recent systematic review^19^. Several studies in this review did not report both the sensitivity and PPV of their models, or provide the confusion matrix such that they could be calculated. One study which did report their model thoroughly, and achieved strong performance, was the model developed by Inselman et al. in the USA^30^. Their model predicted asthma attacks in the six months following discontinuation of biologic therapy and achieved 81% sensitivity and 84% PPV. However, that study focused on a highly selective population: biologics are an often highly effective and safe alternative to oral steroid treatment, but their high cost means that they are typically reserved for those with an extensive history of previous attacks and high ongoing risk^31,32^. Our study was also able to achieve high sensitivity (100%) and PPV (49%) in those with oral corticosteroid prescriptions in the last year, however the specificity was very poor (6%). Furthermore, we posit that while identifying high-risk patients without a history of attacks has proved to be a much more challenging task, these patients may benefit most from health education interventions.

The great potential of EHRs to conduct large-scale, resource efficient, observational research has been well discussed^33,34^. However, the question remains whether EHRs, as they are currently generated, are a viable source of data for predictions about *individual* patients, particularly when it comes to making healthcare decisions on their basis. Firstly, the recording (in coded data) of key features such as diagnosis of comorbidities maybe be poor, particularly in practices where the coding is often conducted by a non-clinical member of staff^35^. It is likely that there is a wealth of useful data already being captured in primary care, but stored in free-text clinical notes^36^, which are rarely available for research due to re-identification risk. Many patients with asthma may have a scarcity of coded historical data: in our dataset 87% of samples were preceded by *at most* one asthma-related consultation in the previous year. In only 2% of samples participants were able to make use of a peak flow recording in the previous two weeks. In the future, linkage between primary care records and smart devices or other patient-sourced data sources may facilitate the leveraging of more regular data for patients who are willing and able to share.

Furthermore, coded primary care diagnoses are an evolving phenomenon, with hypotheses of suspected asthma being examined through formal spirometry tests and observation of outcomes following treatment^37,38^. Asthma diagnosis is a particularly difficult task, due to the heterogeneity of clinical presentation^39^, and the range of conditions with similar symptoms, such as COPD. As such, many previous studies have specifically excluded patients with COPD^19^. In this study, we conducted sensitivity analyses on those with COPD diagnoses recorded at a later date (although the model was not trained on samples from people with a COPD diagnosis at that time) in order to determine whether this was associated with model performance. We found that there was no substantial difference in performance for those with COPD diagnosis recorded within five years of the observation index date, compared to those without COPD ever recorded, however the model had lower sensitivity in those with COPD diagnosis recorded more than five years from their observation date (23% versus 37%). The validity, generalisability, and interpretation of this finding is therefore unclear.

In our analysis, the logistic regression model outperformed the more complex, non-linear algorithms such as the random forest. This may indicate that there were no substantial interactions between model features, and also has the result that the model is much easier to interpret. Further work is warranted to determine whether the model can be improved by removing any of the features, or through the addition of expert-determined interaction terms.

### Strengths and limitations

This study was able to leverage the wealth of longitudinal data from a large, uncontrolled, representative population, covering the whole geography of Scotland^20^. We also used an expert-driven guideline-based operational definition of our outcome^22^, which ensures that it is aligned well with current clinical practice as well as other research studies. We tested four algorithms (Naïve Bayes Classifier, Logistic Regression, Random Forests, and Extreme Gradient Boosting), and a selection of hyperparameters, resulting in twelve different models. We also took care to avoid *leakage* in the model development: the use of data in model training that would not be available in the deployment setting, which overestimates the model’s predictive performance^40^. Specifically, there was no crossover of patients between the training and testing partitions, feature scaling in both the training and testing partitions was based on the values observed in the training data only, and linked data were only used for outcomes and not for the features for prediction.

The main limitation to this study is that the model has very poor sensitivity in those without any history of asthma attacks in the past two years (4%). However, the PPV was 29%, which still demonstrates that the model is able to detect some individuals who may be otherwise considered low risk. Future research should consider explicitly looking to train a model in those with no recent history of attacks, for whom the incidence rate will be very low, but the potential for impact especially large.

Overall, the population definition may have been a hinderance to the model’s predictive ability. This study employed limited exclusion criteria, meaning that this model would be in principle applicable to those diagnosed with asthma across the spectrum of severity. However, the diversity of the population may have presented too large a challenge for the models to learn from. For example, if certain risk factors were only pertinent to specific asthma phenotypes (e.g. seasonality for those with allergic asthma)^41^, the model might not be able to detect this pattern due to limitations in sample size, or model parameters such as the depth of the trees or the number of trees.

Including multiple samples per individual, throughout their years of registration at their general practice, allows us to capture some of the inter-person variability in risk, it also means that some population subgroups will be over-represented in the data. For example, if smokers have more consultations that non-smokers, they will contribute more weight to the model than if there was a single sample per person. Furthermore, the performance measures in this analysis are relational to the number of samples, and as such the performance is skewed by that of those with a larger number of encounters. As shown in Table 4, only 13% of samples were for those with multiple encounters in the previous year, but these individuals also had a higher attack rate (30% vs 4%). The sensitivity and PPV were higher in this sub-cohort (61% vs 18%, and 42% vs 30%, respectively).

## Conclusions

Our model achieved good calibration between predicted risk of an asthma attack in the year following primary care encounter for asthma or respiratory infection and observed rates of attacks. Our implemented binary classification rule flagged approximately 1 in 10 patients as being high risk, and in this group approximately 1 in 3 had asthma attacks, compared to approximately 1 in 16 in the low-risk group. We demonstrated the effect of various model specifications on the balance between minimising false negatives (missed opportunities for intervention) and false positives (inefficient resource allocation), which allow the model to be tailored according to the desired clinical utility. Building on this analysis, there is a need for user-centred research to explore optimal ways of presenting this information to clinicians so it can be incorporated into routine care. There is also the need to identify additional data sources (e.g., pollen, pollution and weather data) that could potentially be incorporated into future iterations of our risk prediction algorithm.

## Key messages

### What is already known on this topic:

- Clinical risk prediction models are increasingly proposed as a solution to improve efficiency and equality of healthcare. The high prevalence and heterogeneity of asthma, combined with the low incidence of serious outcomes, often results in suboptimal self-management, and missed opportunities for clinical intervention. Integration of the model into a primary care clinical decision support tool, developed on routinely collected data, may be the most appropriate route, however risk prediction in this setting remains a challenge.

### What this study adds:

- Our model was able to identify low-risk patients, with a specificity 95% and a negative predictive value of 93%. The high-risk patient group was harder to identify, and the model only achieved 30% sensitivity and 36% positive predictive value; however, the model had good calibration (slope of 1.02). The Area under the Curve was 0.75. The model can be adjusted to fit best clinical need, such as adjusting the classification threshold relative to estimated misclassification costs of a given intervention, focussing on calibration or filtering to a less broad asthma population, depending on the desired clinical utility.

### How this study might affect research, practice or policy:

- This study examines the discrimination and calibration of a developed prediction model of asthma attacks on a 1-year horizon, on a diverse population in a real-world setting. By examining a range of methodological approaches, we highlight a range of possible modifications to the model design to assist with specific clinical tasks, such as health education, and efficient resource utilisation.

## Supplementary information

Supplementary Material

Reporting Guidelines Checklists

## Data Availability

In our study, individual patient data were collected at the practice-level, and individual consent was therefore not obtained. Permissions for the ALHS project were obtained from the South East Scotland Research Ethics Committee 02 [16/SS/0130] and the Public Benefit and Privacy Panel (PBPP) for Health and Social Care [1516-0489]. The ALHS data were held by the National Services Scotland electronic Data Research and Innovation Service (eDRIS) in the National Safe Haven. Applications for data access should be addressed to phs.edris@phs.scot.

## Code availability

Analysis was conducted in R Version 3.6.1, and all R scripts (data cleaning, feature extraction, analysis, and all clinical code lists) will be made freely available upon publication at: https://github.com/hollytibble/Asthma_Attack_Risk_Classifier.

## References

[1] Asthma UK. *UK Asthma Death Rates among Worst in Europe*. (2017).

[2] Rodrigo, G. Asthma in adults (acute). *BMJ Clin. Evid.* **04**, 1513 (2011). PMC3661228 21463536

[3] Martin, M. J., Beasley, R. & Harrison, T. W. Towards a personalised treatment approach for asthma attacks. *Thorax* **75**, 1119–1129 (2020). 32839286 10.1136/thoraxjnl-2020-214692

[4] Bloom, C. I. et al. Exacerbation risk and characterisation of the UK’s asthma population from infants to old age. *Thorax* **73**, 313–320 (2018). 29074814 10.1136/thoraxjnl-2017-210650

[5] Chen, S. et al. Systematic literature review of the clinical, humanistic, and economic burden associated with asthma uncontrolled by GINA Steps 4 or 5 treatment. *Curr. Med. Res. Opin.* **34**, 2075–2088 (2018). 30047292 10.1080/03007995.2018.1505352

[6] Gruffydd-Jones, K., Nicholson, I., Best, L. & Connell, E. Why don’t patients attend the asthma clinic?. *Prim. Care Respir. J.* **7**, 36–38 (1999).

[7] Royal College of Physcians. *Why Asthma Still Kills: The National Review of Asthma Deaths (NRAD)*. www.rcplondon.ac.uk/nrad (2014). 10.3399/bjgp14X682237 PMC4220235 25348975

[8] Pinnock, H. et al. Systematic meta-review of supported self-management for asthma: a healthcare perspective. *BMC Med.* **15**, 64 (2017). 28302126 10.1186/s12916-017-0823-7 PMC5356253

[9] British Thoracic Society/Scottish Intercollegiate Guideline Network. *British Guideline on the Management of Asthma*. (2019).

[10] Levy, M. L. et al. Key recommendations for primary care from the 2022 Global Initiative for Asthma (GINA) update. *npj Prim. Care Respir. Med.* **33**, 1–13 (2023). 36754956 10.1038/s41533-023-00330-1 PMC9907191

[11] McClatchey, K. et al. IMPlementing IMProved Asthma self-management as RouTine (IMP2ART) in primary care: study protocol for a cluster randomised controlled implementation trial. *Trials* **24**, 252 (2023). 37013577 10.1186/s13063-023-07253-9 PMC10068707

[12] Kelsey, T. & Cavendish, W. Personalised health and care 2020: Using data and Technology to Transform Outcomes for Patients and Citizens. A framework for action. *National Information Board* 1–66. 10.1177/0272989X06295361 (2014).

[13] Nwaru, B. I., Friedman, C., Halamka, J. & Sheikh, A. Can learning health systems help organisations deliver personalised care?. *BMC Med.* **15**, 177 (2017). 28965492 10.1186/s12916-017-0935-0 PMC5623976

[14] Xiao, C., Choi, E. & Sun, J. Opportunities and challenges in developing deep learning models using electronic health records data: a systematic review. *J. Am. Med. Inform. Assoc.* **25**, 1419–1428 (2018). 29893864 10.1093/jamia/ocy068 PMC6188527

[15] Adkins, D. E. Machine learning and electronic health records: a paradigm shift. *AJP* **174**, 93–94 (2017). 10.1176/appi.ajp.2016.16101169 PMC5807064 28142275

[16] Fernández, A. et al. *Learning from Imbalanced Data Sets*. 10.1007/978-3-319-98074-4 (2018).

[17] Rahman, M. M. & Davis, D. N. Addressing the class imbalance problem in medical datasets. *Int. J. Mach. Learn. Comput.* **3**, 224–228 (2013).

[18] Hastie, T., Tibshirani, R. & Friedman, J. *Elements of Statistical Learning* (*2nd Edition)*. *Springer Series in Statistics* (2009).

[19] Ma, L. & Tibble, H. Primary care asthma attack prediction models for adults: a systematic review of reported methodologies and outcomes. *J. Asthma Allergy* **17**, 181–194 (2024). 38505397 10.2147/JAA.S445450 PMC10948327

[20] Soyiri, I. N. et al. Improving predictive asthma algorithms with modelled environment data for Scotland: an observational cohort study protocol. *BMJ Open.* **8**, e23289 (2018). 10.1136/bmjopen-2018-023289 PMC5961591 29780034

[21] Tibble, H., Sheikh, A. & Tsanas, A. Derivation of asthma severity from electronic prescription records using British thoracic society treatment steps. *BMC Pulm. Med.* **22**, 397 (2022). 36329425 10.1186/s12890-022-02189-3 PMC9635147

[22] Reddel, H. K. et al. An official American Thoracic Society/European Respiratory Society statement: Asthma control and exacerbations - Standardizing endpoints for clinical asthma trials and clinical practice. *Am. J. Respiratory Crit. Care Med.* **180**, 59–99 (2009). 10.1164/rccm.200801-060ST 19535666

[23] Chawla, N. V., Bowyer, K. W., Hall, L. O. & Kegelmeyer, W. P. SMOTE: synthetic minority over-sampling technique. *J. Artif. Intell. Res.* **16**, 321–357 (2002).

[24] He, H. & Garcia, E. A. Learning from Imbalanced Data. *IEEE Trans. Knowl. Data Eng.* **21**, 1263–1284 (2009).

[25] Kiefer, J. Sequential minimax search for a maximum. In *Proceedings of the American Mathematical Society*10.2307/2032161 (1953).

[26] Tibble, H. et al. Predicting asthma attacks in primary care: protocol for developing a machine learning-based prediction model. *BMJ Open.* **9**, e028375 (2019). 31292179 10.1136/bmjopen-2018-028375 PMC6624024

[27] Kerr, K. F., Meisner, A., Thiessen-Philbrook, H., Coca, S. G. & Parikh, C. R. RiGoR: Reporting guidelines to address common sources of bias in risk model development. *Biomark. Res.* **3**, 2 (2015). 25642328 10.1186/s40364-014-0027-7 PMC4312434

[28] Collins, G. S., Reitsma, J. B., Altman, D. G. & Moons, K. G. Transparent reporting of a multivariable prediction model for individual prognosis or diagnosis (TRIPOD): the TRIPOD Statement. *BMC Med.* **13**, 1 (2015). 25563062 10.1186/s12916-014-0241-z PMC4284921

[29] Benchimol, E. I. et al. The REporting of studies Conducted using Observational Routinely-collected health Data (RECORD) Statement. *PLOS Med.* **12**, e1001885 (2015). 26440803 10.1371/journal.pmed.1001885 PMC4595218

[30] Inselman, J. W. et al. A prediction model for asthma exacerbations after stopping asthma biologics. *Ann Allergy Asthma Immunol.* S1081-120601972-X. 10.1016/j.anai.2022.11.025 (2022). 10.1016/j.anai.2022.11.025 PMC9992017 36509405

[31] Jackson, D. J. et al. Characterisation of patients with severe asthma in the UK Severe Asthma Registry in the biologic era. *Thorax* **76**, 220–227 (2021). 33298582 10.1136/thoraxjnl-2020-215168 PMC7892381

[32] Mansur, A. H. et al. Biologic therapy practices in severe asthma; outcomes from the UK Severe Asthma Registry and survey of specialist opinion. *Clin. Exp. Allergy* **53**, 173–185 (2023). 10.1111/cea.14222 36057784

[33] Ryan, D. et al. Use of electronic medical records and biomarkers to manage risk and resource efficiencies. *Eur. Clin. Respiratory J.* **4**, 1293386 (2017). 10.1080/20018525.2017.1293386 PMC5404653 28469833

[34] Callahan, A., Shah, N. H. & Chen, J. H. Research and reporting considerations for observational studies using electronic health record data. *Ann. Intern. Med.* **172**, S79–S84 (2020). 32479175 10.7326/M19-0873 PMC7413106

[35] Weatherburn, C. J. Data quality in primary care, Scotland. *Scott. Med. J.* **66**, 66–72 (2021). 33615904 10.1177/0036933021995965

[36] Hayward, R. A., Chen, Y., Croft, P. & Jordan, K. P. Presentation of respiratory symptoms prior to diagnosis in general practice: a case–control study examining free text and morbidity codes. *BMJ Open.* **5**, e007355 (2015). 26070795 10.1136/bmjopen-2014-007355 PMC4466603

[37] Daines, L. et al. *Asthma Guidelines in Practice*. https://www.pcrs-uk.org/resource/current/asthma-guidelines-practice-pcrs-consensus (2019).

[38] Daines, L., Lewis, S., Schneider, A., Sheikh, A. & Pinnock, H. Defining high probability when making a diagnosis of asthma in primary care: mixed-methods consensus workshop. *BMJ Open.* **10**, e034559 (2020). 32317260 10.1136/bmjopen-2019-034559 PMC7204930

[39] Daines, L. et al. Deriving and validating an asthma diagnosis prediction model for children and young people in primary care. *Wellcome Open. Res.* **8**, 195 (2023). 37928213 10.12688/wellcomeopenres.19078.2 PMC10622861

[40] Kaufman, S., Rosset, S., Perlich, C. & Stitelman, O. Leakage in data mining: Formulation, detection, and avoidance. *ACM Trans. Knowl. Discov. Data* **6**, 15:1–15:21 (2012).

[41] Ilmarinen, P., Tuomisto, L. E. & Kankaanranta, H. Phenotypes, risk factors, and mechanisms of adult-onset asthma. *Mediators Inflamm.* **2015**, 514868 (2015). 26538828 10.1155/2015/514868 PMC4619972

