# Supplementary material for: Development and validation of a machine learning risk prediction model for asthma attacks in adults in primary care
Source: NPJ Prim Care Respir Med. 2025 Apr 23;35:24. doi: 10.1038/s41533-025-00428-8 (PMC12019439; doi:10.1038/s41533-025-00428-8)
Supplement: Supplementary file 1 — Supplementary Material [file 41533_2025_428_MOESM1_ESM.docx]

# Supplementary Material A: ALHS Data Processing Report

Primary Care Encounters

The Primary care dataset consisted of 11,766,100 Read Code records, for 49,307 unique patients with a clinically confirmed diagnosis of asthma reflected in Read codes, dated between January 1^st^, 2000, and 31^st^ March 2017. Duplicates and records with missing or invalid Read Codes were excluded, leaving 10,284,002 records, for 48,975 unique individuals.

Primary Care Prescriptions

The prescriptions dataset contained 41,433,707 records for 671,304 individuals. Records for medications in BNF chapters outwith 3 (respiratory system), 6 (endocrine system, including steroids), and 12 (including nasal allergy treatments) were excluded, leaving 10,136,547 records for 389,551 individuals. Records that were dated outside of the study period, or with dose directions indicating that the record should be deleted due to an error, were excluded, leaving 9,251,488 records remaining for 358,185 individuals.

Accident and Emergency Presentations

There were 1,080,206 A&E records in the study period, for 360,297 unique individuals, which contained valid presenting complaint text or primary disease code. ICD10 codes “J45” and “J46” (and child codes belonging to these parent classes) were used to identify asthma-related A&E presentations, as well as the keyword *asthma* in their presenting complaint text. In total, 7,205 (0.4%) A&E presentations were flagged as asthma-related, for 4,185 unique individuals, of whom 1828 (43.7%) were in our study population (3568 presentations).

Inpatient Hospital Admissions

This dataset is also known as SMR01. There were 1,668,957 inpatient admission records in the study period, for 343,838 unique individuals. Of these, 21,517 (2.3%, 7,899 individuals) were identified as relating to asthma by the presence of ICD10 code “J45” or “J46”. 3342 of the individuals (42.3%) were in our study population (9803 inpatient admissions).

Mortality

There were 91,022 records of deaths (excluding stillbirths) in the study period, of which 190 (0.2%) had ICD10 code “J45” or “J46” as the primary cause of death. 38 of these occurred in our analysis population, in addition to a further 1512 non-asthma deaths.

Data Linkage

Linkage between data sources was conducted deterministically by the data managers, prior to pseudo-anonymisation, using the Scottish unique patient identifier, known as CHI (the Community Health Index).

Follow-up Time

An individual’s follow-up time began six months after their first identified ICS prescription in the study period, with these six months acting as a run-in period to collect data from the EHR. This constraint was included to account for the longitudinal nature of primary care records, in which a diagnosis might be proposed and tested (such as with response to ICS) and subsequently revised (resulting in no further asthma-related consultations). Follow-up ended with an individual’s death, six months after their last recorded ICS prescription in the study period, or one year before either asthma resolution (cessation of symptoms; Read Code 212G.) or the end of the study period (to allow a full year to observe outcomes).

Turned 18

18^th^ June 2009

Start of Study

1^st^ January 2009

Study-Level

Individual-Level

First Asthma Diagnosis Record

21^st^ January 2008

Death

15^th^ May 2018

First Asthma Controller Prescription in Study Period

1^st^ March 2009

6 months

End of Study

31^st^ March 2017

12 months

Last Asthma Controller Prescription in Study Period

28^th^ November 2016

6 months

Figure 2: Example Timeline Demonstrating Individual Follow-up Date Range

*Notes:*

*The red box on the left-most side indicates a date which constitutes part of the patient inclusion criteria.*

*The blue stars indicate the dates of asthma or respiratory infection related primary care consultations.*

*The green box indicates the date range in which consultations are valid to include in the training and testing dataset. This range begins after A) the individual has turned 18, B) the study has commenced, C) the patient has been diagnosed with asthma, and D) 6 months have elapsed since the first asthma controller prescription in the study period. This range ends before A) the patient has died, B) the year prior to the end of the study period, C) the six months prior to the last asthma controller prescription in the study period. As such, the patient has five consultations which can be used in the study dataset.*

# Supplementary Material B: Features used in the asthma attack prediction model

Notes:

The BTS step categorisation does not perfectly align with the treatment steps recommended in the BTS/SIGN Guidelines ^1^, to encompass all treatment scenarios observed in the data. Primarily, step 0 was created here to categorise those currently not actively treating their asthma (including managing their asthma with reliever inhaler alone). Additionally, the BTS/SIGN step 5 includes maintenance OCS treatment, however as it was not possible to identify the indication for OCS prescriptions, this treatment step was disregarded, and step 4 was considered the top level.

High outliers of the numerical variables (age, reliever medication use, adherence, and controllers dispensed) were recoded as 4 times the median, to one significant figure.

Local area code was recorded using the Nomenclature of Units for Territorial Statistics Level-3 (NUTS-3) codes ^2^. Rurality was quantified using the 6-category Scottish Government Urban Rural Classification Scale ^3^, and socioeconomic status was measured using the Scottish Index of Multiple Deprivation ^4^.

Peak expiratory flow was standardised as the percentage of the best measurement from that individual to date (including that measurement itself, if it was the first or best recorded).

Comorbidity diagnoses were categorised by diagnosis recency using the following categorisation: never, in the past year, one up to five years ago, longer than five years ago.

| Risk Factor | Feature Format | Missing Data Handling |
| --- | --- | --- |
| Age | Positive integer | People with no recorded date of birth were excluded from analyses |
| Sex | Categorical {‘F’, ’M’} | People with no recorded sex were excluded from analyses |
| Socioeconomic Status (SIMD Quintile) | Categorical {1:5, missing} | ‘Missing’ category created |
| NUTS-3 Local Area Code | Categorical {“UKM21”, “UKM22”, “UKM23”,”UKM24”,”UKM25”, “UKM26”, “UKM27”, “UKM28”, “UKM31”, “UKM34”, “UKM35”, “UKM38”, “UKM61”, “UKM62”, “UKM63”} | No missing data |
| Rurality | Categories {1:6, missing} | ‘Missing’ category created |
| Smoking Status | Categories {‘current’, ‘former’, ‘non-smoker’} | Lack of information related to smoking recorded as ‘non-smoker’ – thus it could be more accurately named ‘non-smoker or unknown smoking status’. |
| Average daily reliever inhaler usage (micrograms) over most recent prescription interval | Numerical | No missing data – continuous variable defined by presence or absence of specific prescriptions |
| Peak Expiratory Flow in last week (as percentage of highest recorded) | Categorical {‘>90%’, ‘80-90%’, ‘70-80%’, ‘less than 70%’, ‘missing’} | ‘Missing’ category created if there was no recording in the week prior to the index date |
| BTS/SIGN treatment Step | Ordinal Categorical {0:4} | No missing data - categorical variable defined by presence of specific prescriptions, as described elsewhere ^5^ |
| More than one lower respiratory tract infection in previous calendar year, or current calendar year to date | Binary | No missing data - binary variable defined by presence or absence of recorded data |
| More than one primary care asthma encounter in previous calendar year, or current calendar year to date | Binary | No missing data - binary variable defined by presence or absence of recorded data |
| More than one OCS prescription in previous calendar year, or current calendar year to date | Binary | No missing data - binary variable defined by presence or absence of recorded data |
| Number of asthma controller medications dispensed in the previous calendar year | Positive integer | No missing data – continuous variable defined by presence or absence of specific prescriptions |
| Time Since Last Asthma Attack (Recorded in Primary Care) | Categorical {‘one to two years’, ‘six months up to one year’, ‘three up to six months’, ‘one up to three months’, ‘in the last month’ or ‘none in the last two years’} | No missing data - categorical variable defined by presence of recorded data |
| Adherence: days of supply dispensed divided by interval duration for the last three prescriptions (CSA_3) | Numerical | No missing data – continuous variable defined by presence or absence of specific prescriptions |
| Adherence: the percentage of days in the previous calendar year for which there was medication supply available, assuming that supply from overlapping intervals is not discarded (CMA7_2) | Numerical (range = 0 to 1) | No missing data – continuous variable defined by presence or absence of specific prescriptions |
| Blood eosinophil counts (cells per μL) | Categorical {‘≥400’, ‘<400’, ‘missing’} | ‘Missing’ category created |
| Month | Categorical {‘January’, ’February’, ’March’, ‘April’, ‘May’, ‘June’, ‘July’, ‘August’, ‘September’, ‘October’, ‘November’, ‘December’} | No missing data |
| Rhinitis Diagnosis | Comorbidity Diagnosis Recency Categories (see notes) | No missing data - binary variable defined by presence or absence of recorded diagnosis |
| Eczema Diagnosis | Comorbidity Diagnosis Recency Categories (see notes) | No missing data - binary variable defined by presence or absence of recorded diagnosis |
| Anxiety/Depression Diagnosis | Comorbidity Diagnosis Recency Categories (see notes) | No missing data - binary variable defined by presence or absence of recorded diagnosis |
| Nasal Polyps Diagnosis | Comorbidity Diagnosis Recency Categories (see notes) | No missing data - binary variable defined by presence or absence of recorded diagnosis |
| GERD Diagnosis | Comorbidity Diagnosis Recency Categories (see notes) | No missing data - binary variable defined by presence or absence of recorded diagnosis |
| Corticosteroid Nasal Sprays | Comorbidity Diagnosis Recency Categories (see notes) | No missing data - binary variable defined by presence or absence of recorded diagnosis |
| Obesity | Binary | No missing data - binary variable defined by presence or absence of recorded diagnosis |
| Chronic pulmonary disease | Binary | No missing data - binary variable defined by presence or absence of recorded diagnosis |
| Time since last lower respiratory tract infection | Categorical {‘In the past two weeks’, ‘Between two weeks and up to two months ago’, ‘Between two months and up to six months ago’, ‘Between six months and up to twelve months ago’, ‘Between one year and up to two years ago’, ‘None in the last two years’} | No missing data - categorical variable defined by presence of recorded data |
| Nebulised SABA prescription in the last 90 days | Binary | No missing data – continuous variable defined by presence or absence of specific prescriptions |

# Supplementary Material C: Model Selection Methods

## Training Data Enrichment Methods

The training data variations employed different training enrichment methods, assessing how to best overcome problems in model performance due to low outcome prevalence. In S*ynthetic Minority Over-Sampling TEchinque* (SMOTE) ^6,7^*,* each minor class sample (one observation row of the low prevalence group) is paired with another from its K-nearest minority class neighbours, and feature values are generated from a uniform distribution within the range of the example sample pair. This is repeated a specified number of times for each minor class sample. SMOTEing can also use random under-sampling.

The first figure below shows two features from a dataset represented on a scatter plot, for two population groups (points are darker if there are more samples with the same values). The second figure shows the same dataset, but with additional samples SMOTEd into the minor class. The third figure shows the distribution of these two features for the original samples in the major and minor class, plus the additional SMOTEd samples. The R code for these visualisations is available at <https://github.com/hollytibble/Asthma_Attack_Risk_Classifier>.


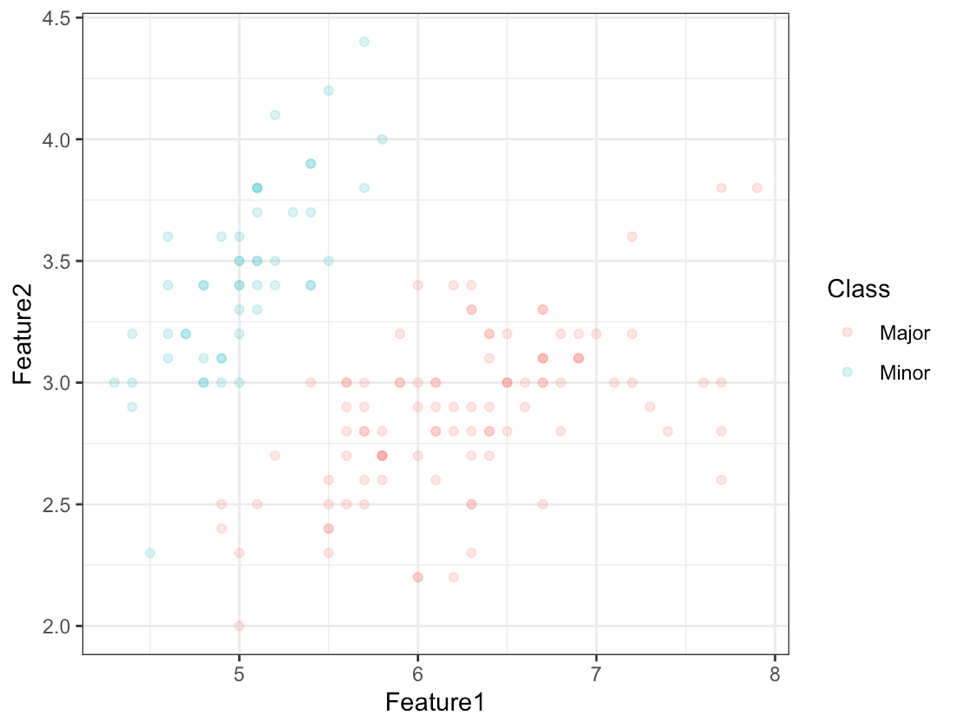

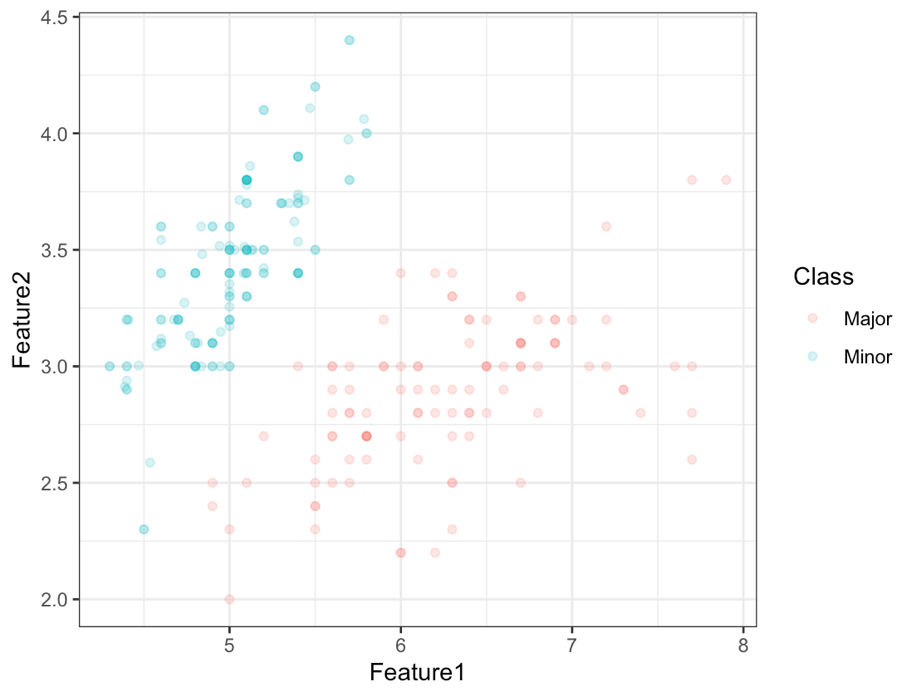

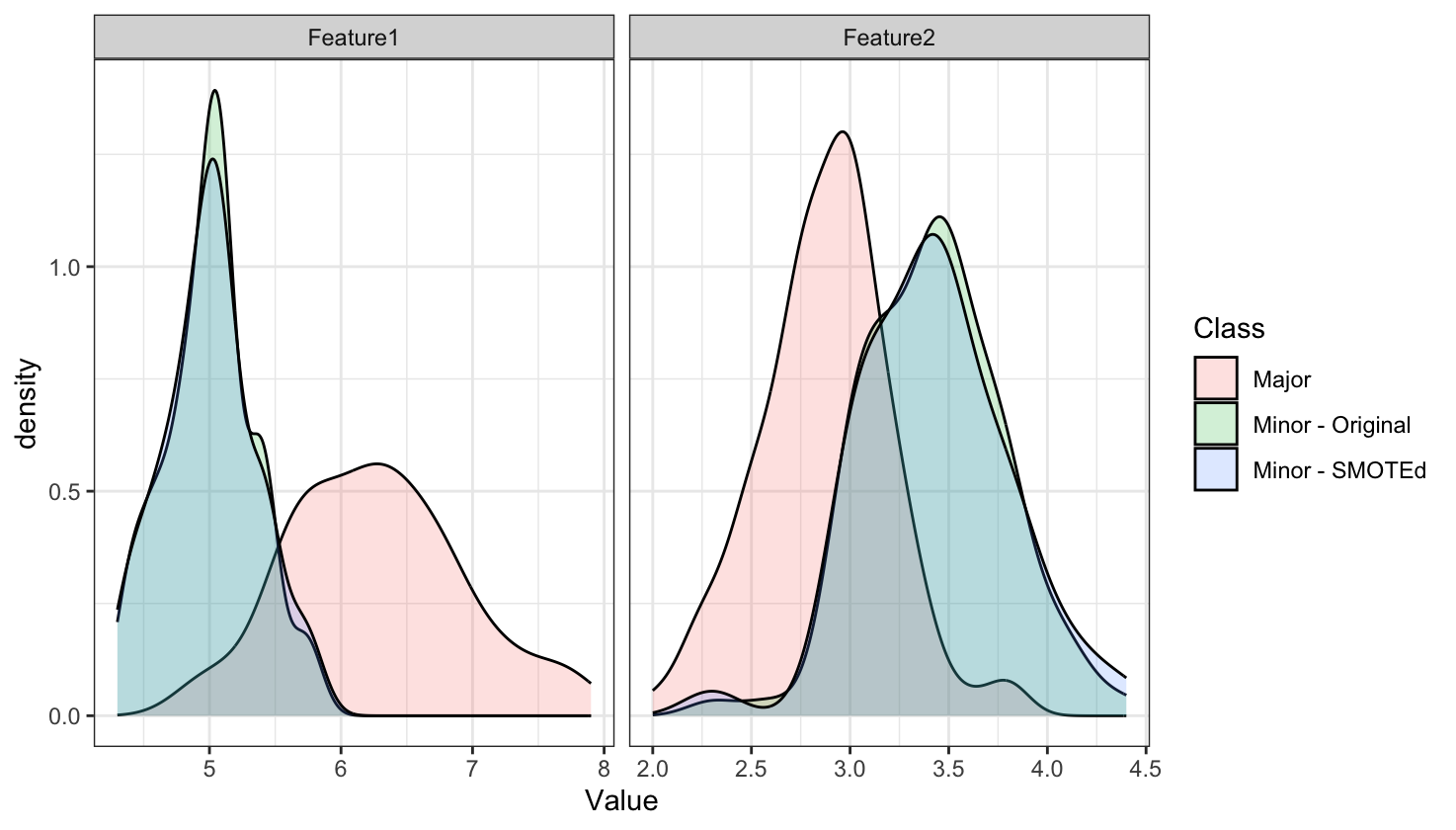


The variations employed herein were: the original training data partition, and the original training data partition with three values of the parameter dup_size (in the function SMOTE from the package smotefamily), representing the desired times of synthetic minority instances over the original number of majority instances: 2, 3, and 4.

The stability of the performance measure estimates across the 10 iterations was evaluated to ensure that there is sufficient confidence in the selection of the model to proceed to the model testing phase, else further iterations will be conducted at this stage. We clarify that only samples from the derivation subset were used to inform SMOTE.

Feature scaling is necessary for SMOTEing, as it is based on the Euclidean distance between minority samples. As such, a sample which was identical in all regards to another except one feature which had a much wider scale than the others may have a higher distance than a sample which was mildly different for all features. As such, feature scaling was conducted on the continuous features by min-max normalisation, independently in the training and testing partitions. Outliers were removed from the continuous features before scaling by right-censoring values at the value of the median, to two significant figures (such as 3200, or 0.32), multiplied by four.

## Model Selection Process

For model selection, the derivation dataset was further randomly partitioned 10 times such that 90% of the samples were used for training (*internal training partition*), and 10% for testing (*internal validation partition*). 12 models were then trained on four variations of the same scaled training data partition, to predict whether an asthma attack would occur in the following twelve weeks (the primary endpoint). The 12 models used the following algorithms: (1) naïve Bayes classifier, (2) logistic regression, (3-6) random forests (with four hyper-parameter values to be optimized) and (7-12) extreme gradient boosting (two hyper-parameter value sets, of size three and two, for a total of six models to be optimized).

## Statistical learning algorithms and hyperparameters

Naïve Bayes Classifier

Implemented using the R function *naivebayes*, from the package of the same name ^9^.

No hyper-parameters.

Logistic Regression

Implemented using the base R function *glm*.

No hyper-parameters.

Random Forests

Implemented using the R function *ranger*, from the package of the same name ^10^.

- *MTRY* = Number of features randomly sampled as candidates at each split (default is the rounded down integer of the square root of the number of features; *k*): floor($\sqrt{k})$, floor(2*$\sqrt{k)}$, floor(4*$\sqrt{k)}$ , floor(8*$\sqrt{k)}$ – in which floor represents the rounded-down integer value.

All other hyper-parameters take implementation default values:

- *NUM.TREES* = Number of trees: 500
- *SAMPLE.FRACTION* = Fraction of cases to sample: 1
- *SPLIT.RULE =* Classification tree splitting rule*: GINI*
- *REGULARIZATION.FACTOR* = Gain penalization:1

Note that the ‘floor’ function denotes the rounded down integer of a value.

Extreme Gradient Boosting

Implemented using the R function *xgboost*, from the package of the same name ^11^.

- *ETA* = Step size shrinkage: 0.05, 0.1, 0.15
- *NROUNDS* = the number of decision trees in the final model: 100, 200

All other hyper-parameters take implementation default values:

- *MAX_DEPTH* = maximum depth of a tree: 6
- *MIN_CHILD_WEIGHT* = minimum sum of instance weight (hessian) needed in a child: 1
- *SUBSAMPLE* = subsample ratio of the training instance: 1
- *COLSAMPLE_BYTREE* = subsample ratio of columns when constructing each tree: 1
- *LAMBDA* = L2 regularization term on weights: 1
- *ALPHA* = L1 regularization term on weights: 0

# Supplementary Material D: Deviations from the published protocol paper

In this appendix, I describe the deviations between my analysis and the original protocol which I had published before commencing the analysis, published in BMJ Open ^12^.

| Topic | Original plan: quote from protocol paper | Revised action |
| --- | --- | --- |
| Inclusion and Exclusion Criteria | “We will identify our study population as all adults (aged 18 and over) with asthma being identified by clinical diagnoses (Read codes), without a chronic obstructive pulmonary disease (COPD) diagnosis, and with relevant prescribing records in primary care. Patients with missing sex or age information will be removed; this and any other patient exclusions from further analysis will be explicitly detailed.” | Those with a diagnosis of Chronic Obstructive Pulmonary Disease (COPD) were identified, and the time between first asthma diagnosis and first COPD diagnosis was estimated. A diagnosis of COPD prior to a diagnosis of asthma excluded patients from primary analyses, however they were retained for a sensitivity analysis (model testing only, no data included in model training). Similarly, for those with a diagnosis of COPD following their asthma diagnosis, the time (and any samples) after their COPD diagnosis was excluded from model training, but was retained for sensitivity analysis (model testing only). |
| Record Left-Censoring | “All records from the derivation dataset (ALHS) will be left-censored on January 2009 in order to align with the primary care prescribing data” | An individual’s follow-up time began six months after their first identified asthma controller medication prescription in the study period (commencing January 2009), with the six months acting as a run-in period to collect data from the EHR. |
| Record Right-Censoring | “All records [will be] right-censored at March 2017, in order to align with the mortality, primary care, and inpatient hospital admission records” | Records were right-censored at the earliest of death, asthma resolution Read code, or one year before the end of the study period (thus, March 2016), to allow a full year for outcomes to be observed. |
| External Validation | “In order to verify that the prediction model performance is not limited to the development dataset and that it generalizes well in new, unseen data … we will evaluate its performance using an external cohort study dataset, the second Seasonal Influenza Vaccination Effectiveness (SIVE II) cohort study …” | Access to the SIVE II dataset was not available for the duration required for analysis to be conducted, due to GDPR requirements for data deletion after the conclusion of the original study. Thus, unfortunately this external validation was not possible to conduct. |
| Topic | Original plan: quote from protocol paper | Revised action |
| Model Features | “Active diagnoses of rhinitis, eczema, gastroesophageal reflux disease (GERD), nasal polyps, and anaphylaxis will be recorded” | A categorical feature was created instead of a binary feature, for the time since the last diagnosis code was recorded, categorised as {‘Never’, ‘In the past year’, ‘One up to five years ago’, ‘Longer than five years ago’}. This allows both recent and past diagnoses to be included. |
|  | N/A | In line with the findings of Price *et al.* ^13^, anxiety and depression were also included (as a single feature, recorded categorically by time since last diagnostic code, as above.) |
|  | N/A | Time since the most recent prescription of nasal spray corticosteroids was included as a risk factor (coded categorically as above). |
|  | N/A | LRTIs (including pneumonia and influenza) were included as risk factors. They were measured by two distinct features: a binary flag for whether more than one had been recorded in the last year (a proxy for susceptibility) and the time since the last recorded infection (to flag periods of recovery). This feature was categorised as: {‘In the past two weeks’, ‘Between two weeks and up to two months ago’, ‘Between two months and up to six months ago’, ‘Between six months and up to twelve months ago’, ‘Between one year and up to two years ago’, ‘None in the last two years’} |
|  | “the number of primary care asthma encounters (days on which at least one asthma related code was recorded) in the previous year will be derived” | A binary feature was created flagging whether or not there were more than one encounter in the previous year. The decision to binarize the data was due to the wide range observed in the counts, which resulted in the differences between lower counts being quashed by the normalisation process. The decision boundary was based on the observed low median number of past encounters observed across the whole analysis population. |

| Topic | Original plan: quote from protocol paper | Revised action |
| --- | --- | --- |
| Model Features | “the prior number of attacks … will be considered time-dependent and accurate at the weekly level.” | As above, this feature was amended to a binary indicator of whether there was more than one asthma attack either in the previous calendar year, or in the current year to date. |
|  | “The mean Short-Acting Beta-2 Agonist (SABA) dose per day will be estimated retroactively by examining the dates between prescriptions” | The mean SABA dose was refined to only include *inhaled* SABA medications, however an additional feature was added to indicate that a nebulised SABA medication had been prescribed in the last 90 days. |
|  | “Adherence to preventer therapy will be approximated using the medication possession ratio, calculated from primary care prescribing records.” | Two measures of adherence were used as risk factors in my prediction model, known as CSA_3 and CMA7_2. The Medication Possession Ratio (MPR; equivalent to the CMA1) was previously selected based on its use by Blakey *et al.* ^14^*,* however upon further investigation the requirement for at least two prescriptions to calculate excluded too many people. |
| Models tested | “… predicting asthma attack occurrence in the following 1, 4, 26, and 52 weeks” | Only the 52-week horizon model was generated. |
|  | “[We will] employ more advanced state of the art principled supervised learning algorithmic tools such as support vector machines…” | It was not feasible to investigate the SVM algorithm due to computational power limitations. |
|  | N/A | We have also included the logistic regression algorithm. |
|  | Random Forest classifier hyper-parameters:   - “NTREE = Number of trees to grow (default 500): 500, 750, 1000   MTRY = Number of variables randomly sampled as candidates at each split (default square root of the number of predictors; k): floor($0.5*\sqrt{k)}$, floor($\sqrt{k})$, floor(2*$\sqrt{k)}$” | For RFs, higher values of mtry (candidate features at each split) were tested (floor($\sqrt{k})$, floor(2*$\sqrt{k)}$, floor(4*$\sqrt{k)}$, and floor(8*$\sqrt{k)}$), but the models with higher numbers of trees (*ntrees*) than the default were removed. More trees generally result in a better variance-bias trade-off, and thus a lower risk of overfitting, but the improvement is not always efficient relative to the increased training time. |
|  | “Implemented using the r function randomForest, from the package of the same name” | The R implementation was changed to the faster *ranger* package. |

| Topic | Original plan: quote from protocol paper | Revised action |
| --- | --- | --- |
| Models Tested | Extreme Gradient Boosting  “Implemented using the r package *xgboost,* with 10-fold cross validation, repeated 3 times.” | For XGBoost, repeated cross-validation was not used, and the hyper-parameters were instead evaluated in the same way as the RFs, for consistency. |
|  | XGBoost classifier hyper-parameters:   - “NROUNDS = maximum number of iterations (default 100): 50,100 - MAXDEPTH = Maximum depth of each tree (default = 6): (1:5)^2   ETA = step size of each boosting step (default = 0.3): 0.25, 0.5, 1” | For XGBoost, lower values of the learning rate (eta) were used (0.05, 0.1, and 0.15, instead of 0.25, 0.5 and 1). Although this increased the computation time, it vastly increased the stability of the model’s performance across iterations, which was important to ensure that the first 10 iterations were sufficient to evaluate the model performance compared to the other algorithms. In line with the lowered learning rate, higher numbers of boosting rounds were tested (100 and 200, rather than 50 and 100). To reduce the number of models being tested, only the default maximum tree depth (6 branches deep) was used. |
| Analysis Plan | “Ensemble: Stacking  Combining models from different classifiers, with an over-arching supervisor model which determines the best way to use all sources of information for prediction. The base set of weak learners will comprise all aforementioned model and hyper-parameter combinations, and the meta-learner (random forest with 500 trees and mtry = floor($0.5*\sqrt{k)}$) will use all weak learners with a validation set performance in the top 50%. “ | Stacking was not employed due to computational power limitations. |

| Topic | Original plan: quote from protocol paper | Revised action |
| --- | --- | --- |
| Analysis Plan | “We will run 100 iterations [of each model] for statistical confidence, each time randomly permuting samples prior to determining the three subsets” (training, testing and validation). | The data partitioning procedure was altered such that instead of running 100 iterations of every model, the model selection process was only based on the first ten iterations. As such, to ensure that there was no overlap between the validation and model selection partitions, a 10% hold-out set was used, and the partitioning in the remaining 90% was changed to 90% training and 10% testing. |
|  | “Performance in the testing datasets will be assessed using the MCC, and the additional metrics of sensitivity, specificity, positive and negative predictive values, and the F_1_ measure” | The F_1_ measure was not reported due to the restriction to 52 weeks as the outcome prediction horizon, however it is still calculable from the confusion matrix provided. |
|  | “Performance in the testing datasets will be assessed using … the Bayesian Information Criterion (BIC) to obtain a trade-off between model complexity and accuracy.” | The BIC was not reported. |

| Topic | Original plan: quote from protocol paper | Revised action |
| --- | --- | --- |
| Analysis Plan | “A selection of training enrichment methods will be trialled, in order to assess how to best overcome poor performance as a result of low outcome prevalence. … As such, this start of this process (the first 20 iterations of training each model) will be repeated five times, using:   1. the original analysis dataset, 2. original data with additional duplicates of the positive outcome records (a method known as over-sampling), 3. original data, with a selection of the negative outcome records removed (under-sampling), 4. original data with additional slightly modified duplicates of the positive outcome records, with a selection of the negative outcome records removed (Synthetic minority over-sampling; SMOTE) 5. original data, using the outcome classification threshold to maximise the primary metric” | Due to the extreme class imbalance, the pure under-sampling and over-sampling approaches would have resulted in either a very low sample size, or a dataset with almost 50% replicated samples, respectively. As such, three SMOTE tests were conducting, using different balanced of the under and over-sampling parameters. For each SMOTE balance choice, a selection of classification thresholds was employed. |
|  | “We will re-train the model using the hyper-parameter specifications from the best performing model, with a modified version of the derivation dataset which incorporates data extracted from secondary care records (such as A&E presentations for asthma attack not captured in primary care records) in the determination of the risk factors. This allows us to evaluate the added value of secondary care data linkage in the prediction of impending asthma attacks, and will be determined by the same metrics used for the primary model evaluation” | This analysis will be reported elsewhere. |

# Supplementary Material E: Reporting Guidelines Checklists

Guidelines Used:

- RiGoR: Reporting Guidelines to address common sources of bias in Risk model development, by Kerr *et al.*  ^15^,
- TRIPOD: Transparent Reporting of a multivariable prediction model for Individual Prognosis Or Diagnosis, by Collins *et al.* ^16^,
- RECORD: Reporting of studies Conducted using Observational Routinely-collected health Data, by Benchimol *et al.* ^17^.

Note: RiGoR items related to participant recruitment (4a and 4b) and Biomarker Data (6 to 8 and 11) were not included as they were not relevant to this study.

| **Section/Topic** | **Checklist**  **(Item Number)** | **Checklist Item** | **Section** |
| --- | --- | --- | --- |
| Title and abstract | | | |
| Title | TRIPOD (1) | Identify the study as developing and/or validating a multivariable prediction model, the target population, and the outcome to be predicted. | Title |
|  | RECORD (1.1) | The type of data used should be specified in the title or abstract. When possible, the name of the databases used should be included. | Title |
|  | RiGoR (1) | Identify the article as reporting the development of a risk model combining multiple predictors (MeSH “Risk”, possibly “risk factor” and/or “biomarker”) | Title |
| Abstract | TRIPOD (2) | Provide a summary of objectives, study design, setting, participants, sample size, predictors, outcome, statistical analysis, results, and conclusions. | Abstract |
|  | RECORD (1.2) | If applicable, the geographic region and timeframe within which the study took place should be reported in the title or abstract. | Abstract |
|  | RECORD (1.3) | If linkage between databases was conducted for the study, this should be clearly stated in the title or abstract. | Abstract |
| Introduction | | | |
| Background and objectives | TRIPOD (3a) | Explain the medical context (including whether diagnostic or prognostic) and rationale for developing or validating the multivariable prediction model, including references to existing models. | Background |
|  | RiGoR (2) | Identify the overarching goal – why would an effective risk model be valuable to clinical care, public health, or research? | Background |
|  | TRIPOD (3b) | Specify the objectives, including whether the study describes the development or validation of the model or both. | Background |
| Methods | | | |
| Source of data | TRIPOD (4a) | Describe the study design or source of data (e.g., randomized trial, cohort, or registry data), separately for the development and validation data sets, if applicable. | Methods: Data |
|  | TRIPOD (4b) | Specify the key study dates, including start of accrual; end of accrual; and, if applicable, end of follow-up. | Methods: Data |
|  | RECORD (12.3) | State whether the study included person-level, institutional-level, or other data linkage across two or more databases. The methods of linkage and methods of linkage quality evaluation should be provided. | Supplementary Material A |
| Participants | TRIPOD (5a), RiGoR (3) | Specify key elements of the study setting (e.g., primary care, secondary care, general population) including number and location of centres. | Methods: Data |
|  | TRIPOD (5b), RECORD (6.1),  RiGoR (3) | Describe eligibility criteria for participants. The methods of study population selection (such as codes or algorithms used to identify subjects) should be listed in detail. If this is not possible, an explanation should be provided. | Methods: Analysis Population,  GitHub repository |
|  | RiGoR (5) | Describe the study design. | Background |
|  | RECORD (6.2) | Any validation studies of the codes or algorithms used to select the population should be referenced. If validation was conducted for this study and not published elsewhere, detailed methods and results should be provided. | Not Applicable |
|  | RECORD (6.3) | If the study involved linkage of databases, consider use of a flow diagram or other graphical display to demonstrate the data linkage process, including the number of individuals with linked data at each stage. | Figure 1 |
|  | TRIPOD (5c) | Give details of treatments received, if relevant. | Not Applicable |
| Outcome | TRIPOD (6a), RiGoR (9) | Clearly define the outcome that is predicted by the prediction model, including how and when assessed. | Methods: Outcome Ascertainment |
|  | TRIPOD (6b) | Report any actions to blind assessment of the outcome to be predicted. | Not Applicable |
|  | RECORD (7.1) | A complete list of codes and algorithms used to classify exposures, outcomes, confounders, and effect modifiers should be provided. If these cannot be reported, an explanation should be provided. | GitHub repository |
| Predictors | TRIPOD (7a), RiGoR (12a) | Clearly define all predictors used in developing or validating the multivariable prediction model, including how and when they were measured. | Supplementary Material B,  GitHub repository |
|  | TRIPOD (7b) | Report any actions to blind assessment of predictors for the outcome and other predictors. | Not Applicable |
|  | RiGoR (15) | For multi-center studies with the possibility of confounding by center, describe methods for adjusting or accounting for center effects. | Methods: Analysis Population |
| Sample size | TRIPOD (8) | Explain how the study size was arrived at. | Supplementary Material A,  Figure 4 |
| Missing data | TRIPOD (9), RiGoR (16) | Describe how missing data were handled (e.g., complete-case analysis, single imputation, multiple imputation) with details of any imputation method. | Methods: Analysis Population,  Supplementary Material B. |
| Statistical analysis methods | TRIPOD (10a) | Describe how predictors were handled in the analyses. | Supplementary Material B,  GitHub repository |
|  | TRIPOD (10b), RiGoR (12b) | Specify type of model, all model-building procedures (including any predictor selection), and method for internal validation. | Methods: Analysis Plan,  Supplementary Material C |
|  | RiGoR (13) | Document methodology used to develop risk model or classifier | Methods: Analysis Plan,  Supplementary Material C,  Supplementary Material G |
|  | RiGoR (12c) | Describe how model-selection bias was addressed in assessing the performance of final reported model(s). If model-selection bias was not addressed, state this explicitly. | Methods: Analysis Plan |
|  | TRIPOD (10d), RiGoR (10) | Specify all measures used to assess model performance and, if relevant, to compare multiple models. | Methods: Analysis Plan |
|  | RiGoR (14a) | Document methodology to avoid or correct for resubsitution bias in measures of the performance of the final reported model(s). | Methods: Analysis Plan |
|  | RiGoR (14b, 14c) | If an independent validation “test” dataset was used, document that the test data were not used for any part of model development, including variable selection. Document that these data were accessed only when models were finalized. Report the number of models evaluated on the “test” data and how these were selected. If cross-validation was used, state how final reported model was derived. | Methods: Analysis Plan |
|  | RiGoR (17) | Describe methods for assessing model calibration | Methods: Analysis Plan |
| Risk groups | TRIPOD (11) | Provide details on how risk groups were created, if done. | Methods: Analysis Plan |
| Data access and  cleaning methods | RECORD (12.1) | Authors should describe the extent to which the investigators had access to the database population used to create the study population. | Methods: Data Management, Ethics, and Reporting |
|  | RECORD (12.2) | Authors should provide information on the data cleaning methods used in the study. | Supplementary Material B,  GitHub repository |
|  | RECORD (22.1) | Authors should provide information on how to access any supplemental information such as the study protocol, raw data, or programming code. | Methods: Data Management, Ethics, and Reporting |
| Results | | | |
| Participants | TRIPOD (13a), RECORD (13.1) | Describe the flow of participants through the study, including the number of participants with and without the outcome and, if applicable, a summary of the follow-up time. A diagram may be helpful. | Results: Analysis Population,  SupplementaryMaterial A,  Figure 1,  Figure 2,  Figure 4 |
|  | TRIPOD (13b), RiGoR (18) | Describe the characteristics of the participants (basic demographics, clinical features, available predictors), including the number of participants with missing data for predictors and outcome. | Table 1,  Supplementary Material F |
| Model development | TRIPOD (14a) | Specify the number of participants and outcome events in each analysis. | Results: Analysis Population |
|  | TRIPOD (14b) | If done, report the unadjusted association between each candidate predictor and outcome. | Not Applicable |
| Model specification | TRIPOD (15a), RiGoR (19) | Present the full prediction model to allow predictions for individuals (i.e., all regression coefficients, and model intercept or baseline survival at a given time point). | Not Applicable |
|  | TRIPOD (15b) | Explain how to the use the prediction model. | Not Applicable |
| Model performance | TRIPOD (16), RiGoR (20) | Report performance measures (with CIs) for the prediction model. | Not Applicable |
| Model Calibration | RiGoR (21) | Assess and report evidence of risk model calibration | Results: Discrimination and Calibration in Population Subgroups |
| Discussion | | | |
| Limitations | TRIPOD (18), RiGoR (23) | Discuss any limitations of the study (such as nonrepresentative sample, few events per predictor, missing data). | Discussion: Strengths and Limitations |
|  | RECORD (19.1) | Discuss the implications of using data that were not created or collected to answer the specific research question(s). Include discussion of misclassification bias, unmeasured confounding, missing data, and changing eligibility over time, as they pertain to the study being reported. | Discussion: Results in Context |
| Interpretation | TRIPOD (19b) | Give an overall interpretation of the results, considering objectives, limitations, and results from similar studies, and other relevant evidence. | Discussion: Results in Context |
| Implications | TRIPOD (20), RiGoR (22) | Discuss the potential clinical use of the model and implications for future research. | Discussion: Results in Context |
| Other information | | | |
| Supplementary information | TRIPOD (21) | Provide information about the availability of supplementary resources, such as study protocol, Web calculator, and data sets. | Methods: Analysis Plan,  Methods: Data Management, Ethics, and Reporting |
| Funding | TRIPOD (22) | Give the source of funding and the role of the funders for the present study. | Funding |

# Supplementary Material F: Demographics of the ALHS analysis Samples

| **Characteristics** | | **Training Data Samples (N=584,288)** | **Testing Data Samples**  **(N=65,985)** | **COPD-Overlap Samples (N=54,206)** |
| --- | --- | --- | --- | --- |
| Prevalence of Asthma Attacks within One Year of Observation | | | | |
|  | | 46921 (8.03%) | 5842 (8.85%) | 7954 (14.67%) |
| Baseline Age ^a^ | | | | |
|  | 18 to 35 | 120049 (20.55%) | 14583 (22.1%) | 145 (0.27%) |
|  | 36 to 45 | 99763 (17.07%) | 11392 (17.26%) | 2036 (3.76%) |
|  | 46 to 60 | 177474 (30.37%) | 19612 (29.72%) | 12345 (22.77%) |
|  | 61 to 75 | 133041 (22.77%) | 14548 (22.05%) | 24813 (45.78%) |
|  | 76 to 99 | 53961 (9.24%) | 5850 (8.87%) | 14867 (27.43%) |
| Sex | | | | |
|  | Male | 229689 (39.31%) | 26070 (39.51%) | 21832 (40.28%) |
|  | Female | 354599 (60.69%) | 39915 (60.49%) | 32374 (59.72%) |
| Baseline Scottish Index of Multiple Deprivation | | | | |
|  | 1 (Highest Deprivation) | 133653 (22.87%) | 16358 (24.79%) | 14342 (26.46%) |
|  | 2 | 123952 (21.21%) | 14244 (21.59%) | 14186 (26.17%) |
|  | 3 | 99345 (17%) | 11453 (17.36%) | 9099 (16.79%) |
|  | 4 | 123605 (21.15%) | 12882 (19.52%) | 9713 (17.92%) |
|  | 5 (Lowest Deprivation) | 91021 (15.58%) | 9659 (14.64%) | 5927 (10.93%) |
|  | Missing | 12712 (2.18%) | 1389 (2.11%) | 939 (1.73%) |
| Baseline Scottish Urban Rural Classification | | | | |
|  | 1 (Large Urban) | 179754 (30.76%) | 20187 (30.59%) | 14411 (26.59%) |
|  | 2 (Other Urban Area) | 220999 (37.82%) | 24915 (37.76%) | 21634 (39.91%) |
|  | 3 (Accessible Small Towns) | 50455 (8.64%) | 6817 (10.33%) | 7061 (13.03%) |
|  | 4 (Remote Small Towns) | 21352 (3.65%) | 1858 (2.82%) | 1673 (3.09%) |
|  | 5 (Accessible Rural) | 63762 (10.91%) | 6438 (9.76%) | 5440 (10.04%) |
|  | 6 (Remote Rural) | 31567 (5.4%) | 3929 (5.95%) | 2831 (5.22%) |
|  | Missing | 16399 (2.81%) | 1841 (2.79%) | 1156 (2.13%) |
| Baseline BTS Treatment Step | | | | |
|  | 0 (No controllers) | 37252 (6.38%) | 4498 (6.82%) | 4639 (8.56%) |
|  | 1 | 126908 (21.72%) | 14325 (21.71%) | 1124 (2.07%) |
|  | 2 | 56361 (9.65%) | 6375 (9.66%) | 2765 (5.1%) |
|  | 3 | 173151 (29.63%) | 18198 (27.58%) | 7795 (14.38%) |
|  | 4 | 190616 (32.62%) | 22589 (34.23%) | 37883 (69.89%) |
| Baseline Comorbidities ^b^ | | | | |
|  | Rhinitis | 12291 (2.1%) | 1392 (2.11%) | 349 (0.64%) |
|  | Nasal Polyps | 3098 (0.53%) | 206 (0.31%) | 433 (0.63%) |
|  | GERD ^c^ | 7066 (1.21%) | 585 (0.89%) | 1464 (2.7%) |
|  | Eczema | 18992 (3.25%) | 2235 (3.39 %) | 2451 (4.452) |
|  | Chronic pulmonary disease | 5587 (0.96%) | 552 (0.84%) | 2207 (4.07%) |
|  | Anxiety/Depression | 56759 (9.71%) | 6200 (9.40%) | 7072 (13.05%) |
|  | Obesity | 155769 (26.66%) | 18355 (27.82%) | 19693 (36.33%) |

*Notes:*

1. *Age categorisation is presented in this table for ease of viewing, however continuous values are used in the model.*
2. *Diagnoses of eczema, rhinitis, nasal polyps, and anxiety and/or depression in the last five years were included in this table.*
3. *Diagnoses of Gastro-Esophageal Reflux Disease (GERD) in the last year were included in this table.*

# Supplementary Material G: Model Selection Performance Measure Plots

Across enrichment methods, the logistic regression algorithm consistently performed higher than the other algorithms according to the AUC (averaged over all iterations and hyper-parameters investigated) in the ten first internal validation partitions. Furthermore, SMOTEing (enrichment methods 2-4) showed no substantial improvement for any algorithm over the original data.

Figure Notes:

Algorithms: GLM = Generalised Linear Model (Logistic Regression), NBC = Naïve Bayes Classification, RF = Random Forest, XGBoost = eXtreme Gradient Boosting.

Enrichment methods: (1) unenriched data, (2) high up-sampling SMOTE, (3) medium up-sampling SMOTE, (4) low up-sampling SMOTE.

Thresholds: Fixed = 0.5, Variable = Matthews Correlation Coefficient optimising threshold in training data, Prevalence = Prevalence of Outcomes, Balanced = Mean of Prevalence and Variable.


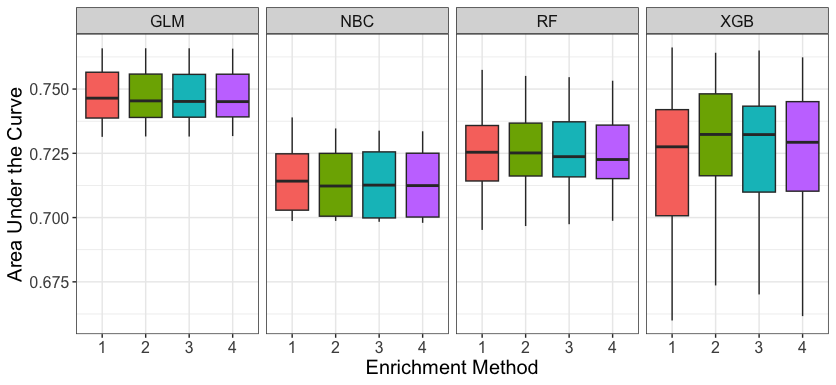


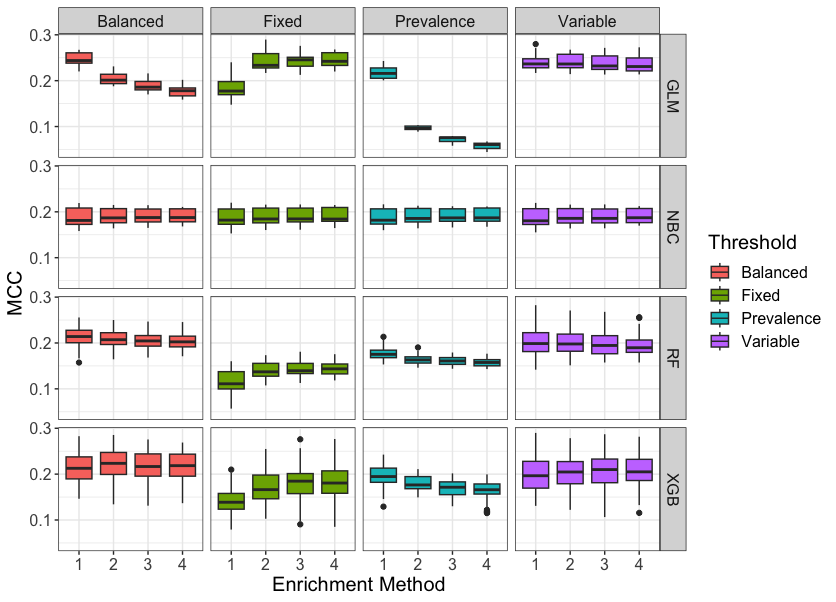


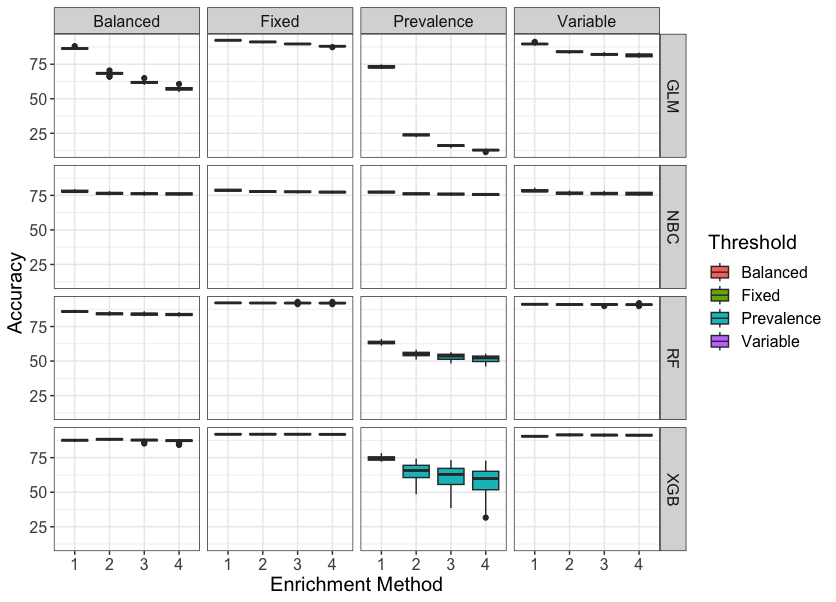


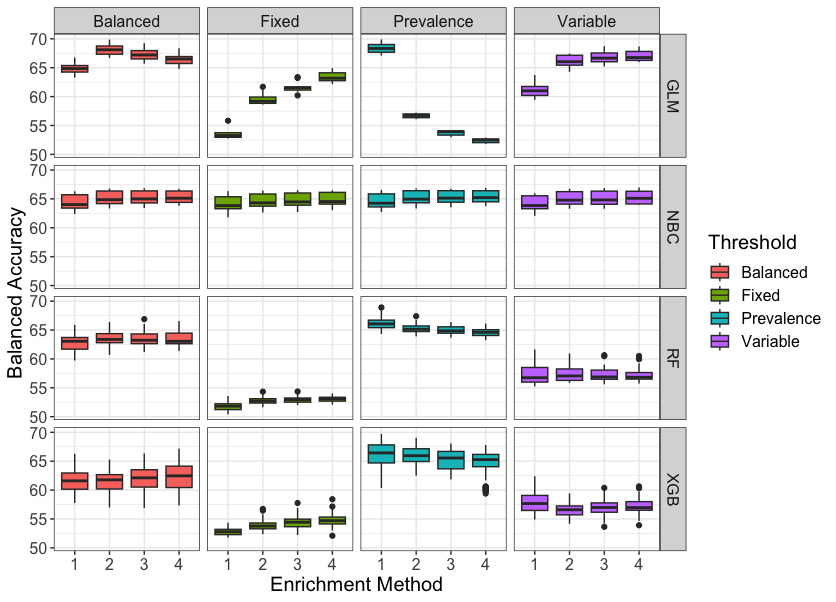


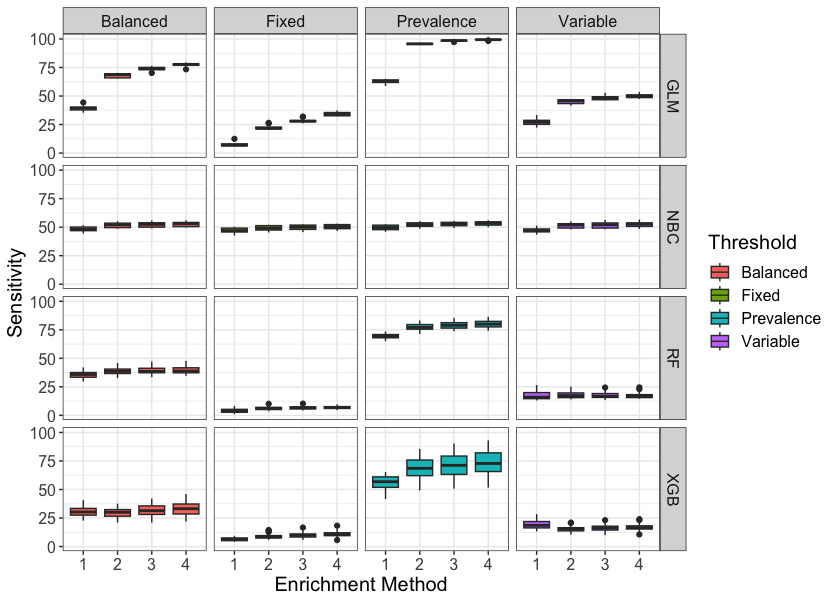


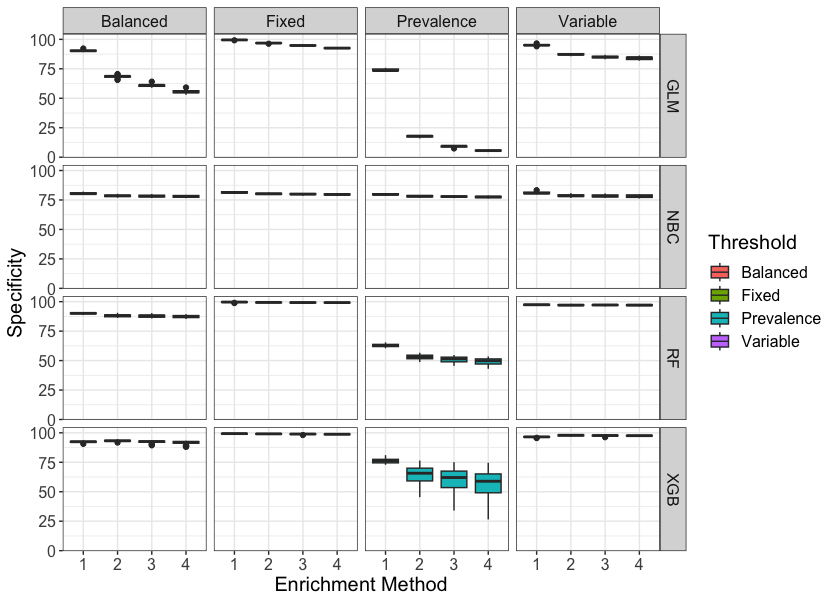


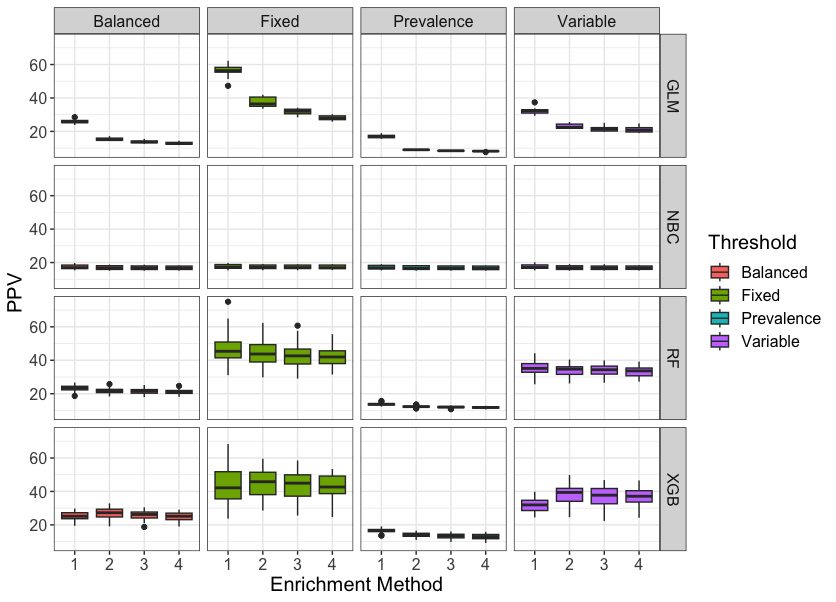


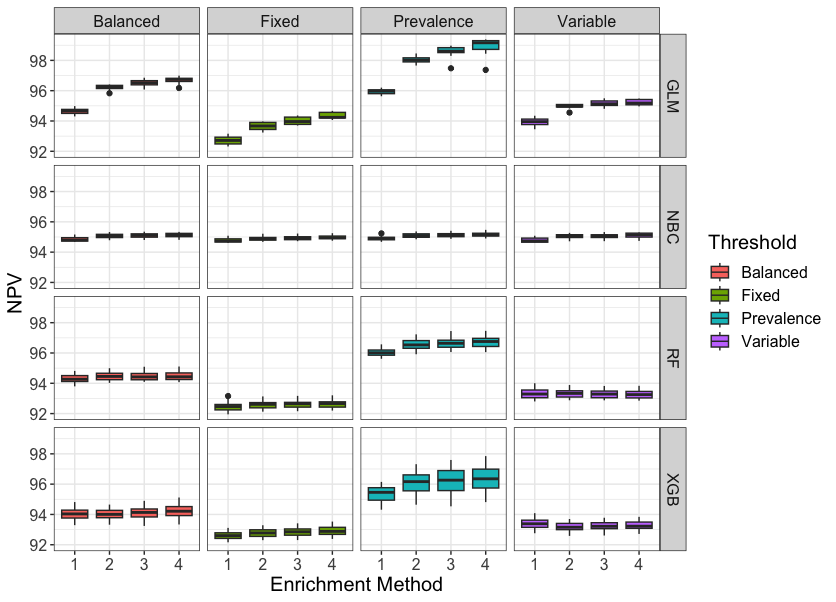


# Supplementary Material H: Model Odds Ratios across derivation data partitioning (100 partitions) and Full Model.

| Feature | 100 Internal Testing Iterations | | | | Full Model Odds Ratio | Full Model p value | |  |
| --- | --- | --- | --- | --- | --- | --- | --- | --- |
|  | Mean Model Odds Ratio | Minimum Model Odds Ratio | Maximum Model Odds Ratio | % of Iteration p<0.05 |  |  |  |  |
| Intercept | 0.032 | 0.026 | 0.038 | 100 | 0.031 | | <0.001 |  |
| Anxiety/Depression Diagnosis = Never | {reference} | | | | | | |  |
| Anxiety/Depression Diagnosis = In the last year | 1.344 | 1.265 | 1.443 | 100 | 1.344 | <0.001 | |  |
| Anxiety/Depression Diagnosis = In the last 2 - 5 years | 1.142 | 1.067 | 1.207 | 100 | 1.142 | <0.001 | |  |
| Anxiety/Depression Diagnosis = Longer than five years ago | 0.488 | 0.227 | 0.655 | 100 | 0.476 | <0.001 | |  |
| BTS/SIGN treatment Step | 1.172 | 1.157 | 1.184 | 100 | 1.171 | <0.001 | |  |
| Blood eosinophil counts (cells per μL) = Missing | {reference} | | | |  |  | |  |
| Blood eosinophil counts (cells per μL) = Greater than or equal to 400 | 1.128 | 1.054 | 1.187 | 100 | 1.127 | <0.001 | |  |
| Blood eosinophil counts (cells per μL) = Less than 400 | 0.978 | 0.92 | 1.017 | 33 | 0.978 | 0.107 | |  |
| Adherence: the percentage of days in the previous calendar year for which there was medication supply available, assuming that supply from overlapping intervals is not discarded (CMA7_2) | 1.104 | 1.05 | 1.168 | 100 | 1.107 | <0.001 | |  |
| Adherence: days of supply dispensed divided by interval duration for the last three prescriptions (CSA_3) | 0.803 | 0.757 | 0.862 | 100 | 0.800 | <0.001 | |  |
| Diagnosis of another chronic pulmonary disease, excluding COPD | 1.656 | 1.346 | 1.836 | 100 | 1.657 | <0.001 | |  |
| Eczema Diagnosis = Never | {reference} | | | |  |  | |  |
| Eczema Diagnosis = In the last year | 1.159 | 1.071 | 1.251 | 99 | 1.161 | <0.001 | |  |
| Eczema Diagnosis = In the last 2 - 5 years | 0.964 | 0.898 | 1.051 | 18 | 0.966 | 0.290 | |  |
| Eczema Diagnosis = Longer than five years ago | 0.336 | 0.000 | 0.585 | 61 | 0.332 | 0.033 | |  |
| GERD Diagnosis = Never | {reference} | | | |  |  | |  |
| GERD Diagnosis = In the last year | 1.157 | 1.018 | 1.252 | 91 | 1.157 | <0.001 | |  |
| GERD Diagnosis = In the last 2 - 5 years | 0.740 | 0.655 | 0.807 | 100 | 0.739 | <0.001 | |  |
| GERD Diagnosis = Longer than five years ago | 0.393 | 0.130 | 0.561 | 100 | 0.387 | 0.001 | |  |
| Nebulised SABA prescription in the last 90 days | 1.916 | 1.846 | 1.999 | 100 | 1.914 | <0.001 | |  |
| NUTS-3 Local Area Code UKM21 | 0.259 | 0.181 | 0.311 | 100 | 0.256 | <0.001 | |  |
| NUTS-3 Local Area Code UKM22 | 0.772 | 0.638 | 0.874 | 100 | 0.778 | <0.001 | |  |
| NUTS-3 Local Area Code UKM23 | 0.738 | 0.586 | 0.865 | 100 | 0.736 | <0.001 | |  |
| NUTS-3 Local Area Code UKM24 | 1.945 | 1.585 | 2.323 | 100 | 1.950 | <0.001 | |  |
| NUTS-3 Local Area Code UKM25 | {reference} | | | |  |  | |  |
| NUTS-3 Local Area Code UKM26 | 1.064 | 0.892 | 1.23 | 33 | 1.071 | 0.135 | |  |
| NUTS-3 Local Area Code UKM27 | 1.379 | 1.137 | 1.584 | 100 | 1.387 | <0.001 | |  |
| NUTS-3 Local Area Code UKM28 | 0.971 | 0.779 | 1.135 | 19 | 0.972 | 0.560 | |  |
| NUTS-3 Local Area Code UKM31 | 1.504 | 1.2 | 1.732 | 100 | 1.508 | <0.001 | |  |
| NUTS-3 Local Area Code UKM34 | 1.396 | 1.314 | 1.566 | 100 | 1.391 | <0.001 | |  |
| NUTS-3 Local Area Code UKM35 | 1.203 | 1.089 | 1.317 | 100 | 1.202 | <0.001 | |  |
| NUTS-3 Local Area Code UKM36 | 2.560 | 0 | 4.236 | 85 | 2.737 | <0.001 | |  |
| NUTS-3 Local Area Code UKM38 | 1.463 | 1.067 | 1.657 | 99 | 1.451 | <0.001 | |  |
| NUTS-3 Local Area Code UKM61 | 1.410 | 1.175 | 1.616 | 100 | 1.409 | <0.001 | |  |
| NUTS-3 Local Area Code UKM62 | 0.826 | 0.653 | 0.973 | 82 | 0.829 | 0.002 | |  |
| NUTS-3 Local Area Code UKM63 | 1.137 | 0.923 | 1.352 | 42 | 1.139 | 0.054 | |  |
| Nasal Polyps Diagnosis = Never | {reference} | | | |  |  | |  |
| Nasal Polyps Diagnosis = In last year | 1.706 | 1.33 | 1.974 | 100 | 1.689 | <0.001 | |  |
| Nasal Polyps Diagnosis = In the last 2 - 5 years | 0.678 | 0.489 | 0.787 | 100 | 0.662 | <0.001 | |  |
| Nasal Polyps Diagnosis = Longer than five years ago | 0.000 | 0.000 | 0.000 | 0 | 0.000 | 0.763 | |  |
| Corticosteroid Nasal Sprays = Never prescribed | {reference} | | | |  |  | |  |
| Corticosteroid Nasal Sprays = Prescribed in the last year | 0.971 | 0.934 | 1.018 | 51 | 0.972 | 0.039 | |  |
| Corticosteroid Nasal Sprays = Prescribed in the last 2 - 5 years | 0.932 | 0.881 | 0.985 | 91 | 0.931 | <0.001 | |  |
| Corticosteroid Nasal Sprays = Prescribed longer than five years ago | 0.501 | 0.303 | 0.619 | 100 | 0.511 | <0.001 | |  |
| Obesity | 1.099 | 1.057 | 1.141 | 100 | 1.100 | <0.001 | |  |
| Peak Expiratory Flow in last week (as percentage of highest recorded) = Missing | {reference} | | | |  |  | |  |
| Peak Expiratory Flow in last week (as percentage of highest recorded) = Less than 70% | 1.124 | 0.943 | 1.33 | 0 | 1.137 | 0.447 | |  |
| Peak Expiratory Flow in last week (as percentage of highest recorded) = 70-80% | 1.350 | 1.137 | 1.483 | 90 | 1.349 | 0.011 | |  |
| Peak Expiratory Flow in last week (as percentage of highest recorded) = 80-90% | 1.057 | 0.987 | 1.154 | 0 | 1.051 | 0.551 | |  |
| Peak Expiratory Flow in last week (as percentage of highest recorded) = 90+% | 0.880 | 0.855 | 0.909 | 100 | 0.881 | <0.001 | |  |
| Rhinitis Diagnosis = Never | {reference} | | | |  |  | |  |
| Rhinitis Diagnosis = In the last year | 0.828 | 0.7 | 0.906 | 97 | 0.830 | <0.001 | |  |
| Rhinitis Diagnosis = In the last 2 - 5 years | 0.610 | 0.526 | 0.685 | 100 | 0.610 | <0.001 | |  |
| Rhinitis Diagnosis = Longer than five years ago | 0.453 | 0.061 | 0.724 | 94 | 0.459 | 0.002 | |  |
| Socioeconomic Status SIMD Quintile = 1 | 1.109 | 1.027 | 1.183 | 98 | 1.112 | <0.001 | |  |
| Socioeconomic Status SIMD Quintile = 2 | 1.088 | 1.014 | 1.148 | 97 | 1.091 | <0.001 | |  |
| Socioeconomic Status SIMD Quintile = 3 | {reference} | | | |  |  | |  |
| Socioeconomic Status SIMD Quintile = 4 | 0.911 | 0.848 | 0.974 | 99 | 0.910 | <0.001 | |  |
| Socioeconomic Status SIMD Quintile = 5 | 0.862 | 0.819 | 0.907 | 100 | 0.864 | <0.001 | |  |
| Socioeconomic Status SIMD Quintile = Missing | {Omitted due to high correlation with Missing UR6} | | | |  |  | |  |
| Sex = Male | {reference} | | | |  |  | |  |
| Sex = Female | 1.364 | 1.314 | 1.434 | 100 | 1.366 | <0.001 | |  |
| Smoking Status = Never | {reference} | | | |  |  | |  |
| Smoking Status = Former | 1.237 | 1.172 | 1.274 | 100 | 1.237 | <0.001 | |  |
| Smoking Status = Current | 1.426 | 1.365 | 1.474 | 100 | 1.422 | <0.001 | |  |
| UR6 Rurality Level = Missing | 0.753 | 0.64 | 0.865 | 100 | 0.752 | <0.001 | |  |
| UR6 Rurality Level 1 (Large Urban) | 0.580 | 0.478 | 0.692 | 100 | 0.583 | <0.001 | |  |
| UR6 Rurality Level 2 (Other Urban Area) | 0.688 | 0.646 | 0.761 | 100 | 0.686 | <0.001 | |  |
| UR6 Rurality Level 3 (Accessible Small Towns) | {reference} | | | |  |  | |  |
| UR6 Rurality Level 4 (Remote Small Towns) | 0.726 | 0.599 | 0.861 | 100 | 0.725 | <0.001 | |  |
| UR6 Rurality Level 5 (Accessible Rural) | 0.725 | 0.673 | 0.784 | 100 | 0.725 | <0.001 | |  |
| UR6 Rurality Level 6 (Remote Rural) | 0.569 | 0.495 | 0.653 | 100 | 0.571 | <0.001 | |  |
| Age | 0.536 | 0.474 | 0.619 | 100 | 0.534 | <0.001 | |  |
| Number of asthma controller medications dispensed in the previous calendar year | 0.829 | 0.765 | 0.891 | 100 | 0.830 | <0.001 | |  |
| Time since last lower respiratory tract infection = Less than two weeks | 2.019 | 1.89 | 2.123 | 100 | 2.020 | <0.001 | |  |
| Time since last lower respiratory tract infection = Two weeks to two months | 1.751 | 1.635 | 1.86 | 100 | 1.749 | <0.001 | |  |
| Time since last lower respiratory tract infection = Two to six months | 1.592 | 1.49 | 1.677 | 100 | 1.592 | <0.001 | |  |
| Time since last lower respiratory tract infection = Six to twelve months | 1.448 | 1.336 | 1.533 | 100 | 1.448 | <0.001 | |  |
| Time since last lower respiratory tract infection = One to two years | 1.494 | 1.412 | 1.596 | 100 | 1.493 | <0.001 | |  |
| Time since last lower respiratory tract infection = More than two years, or unknown | {reference} | | | |  |  | |  |
| Time Since Last Asthma Attack (Recorded in Primary Care) = Less than one month | 3.204 | 3.048 | 3.363 | 100 | 3.206 | <0.001 | |  |
| Time Since Last Asthma Attack (Recorded in Primary Care) = one to three months | 2.986 | 2.81 | 3.132 | 100 | 2.989 | <0.001 | |  |
| Time Since Last Asthma Attack (Recorded in Primary Care) = Three to six months | 2.611 | 2.463 | 2.76 | 100 | 2.612 | <0.001 | |  |
| Time Since Last Asthma Attack (Recorded in Primary Care) = six to twelve months | 2.314 | 2.204 | 2.422 | 100 | 2.312 | <0.001 | |  |
| Time Since Last Asthma Attack (Recorded in Primary Care) = one to two years | 1.959 | 1.869 | 2.082 | 100 | 1.958 | <0.001 | |  |
| Time Since Last Asthma Attack (Recorded in Primary Care) = More than 2 years, or unknown | {reference} | | | |  |  | |  |
| More than one lower respiratory tract infection in previous calendar year, or current calendar year to date | 1.232 | 1.136 | 1.322 | 100 | 1.235 | <0.001 | |  |
| More than one primary care asthma encounter in previous calendar year, or current calendar year to date | 1.274 | 1.237 | 1.319 | 100 | 1.273 | <0.001 | |  |
| More than one OCS prescription in previous calendar year, or current calendar year to date | 2.084 | 1.874 | 2.297 | 100 | 2.085 | <0.001 | |  |
| Average daily reliever inhaler usage (micrograms) over most recent prescription interval | 1.991 | 1.854 | 2.077 | 100 | 1.998 | <0.001 | |  |
| Month = January | 1.065 | 1.042 | 1.089 | 95 | 1.064 | 0.011 | |  |
| Month = February | 1.014 | 0.993 | 1.037 | 0 | 1.014 | 0.588 | |  |
| Month = March | 0.992 | 0.974 | 1.007 | 0 | 0.992 | 0.749 | |  |
| Month = April | 1.184 | 1.155 | 1.207 | 100 | 1.184 | <0.001 | |  |
| Month = May | 1.176 | 1.153 | 1.194 | 100 | 1.177 | <0.001 | |  |
| Month = June | 1.175 | 1.153 | 1.197 | 100 | 1.175 | <0.001 | |  |
| Month = July | 1.096 | 1.075 | 1.12 | 100 | 1.095 | <0.001 | |  |
| Month = August | 1.135 | 1.108 | 1.155 | 100 | 1.135 | <0.001 | |  |
| Month = September | 1.085 | 1.065 | 1.111 | 100 | 1.084 | 0.001 | |  |
| Month = October | 1.109 | 1.084 | 1.128 | 100 | 1.110 | <0.001 | |  |
| Month = November | 1.040 | 1.026 | 1.068 | 5 | 1.041 | 0.103 | |  |
| Month = December | {reference} | | | |  |  | |  |

# Supplementary Material I: Model Calibration Plots for Population Subgroups

Concurrent Chronic Pulmonary Disease, excluding COPD

Blue = yes, red = no


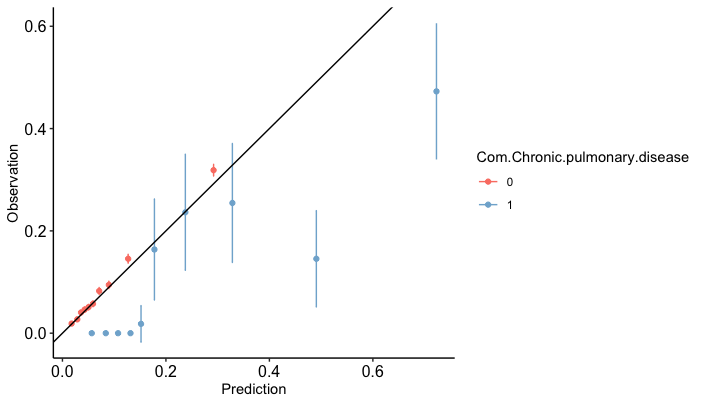


BTS/SIGN Treatment Step

Red = 0, Blue = 1, Orange = 2, Green = 3, Pink = 4


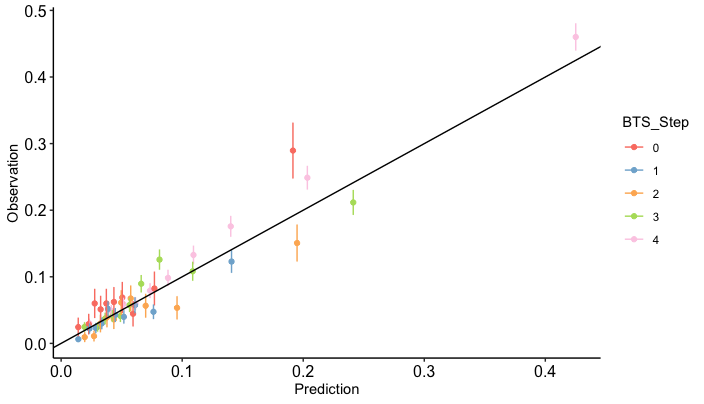


Peak Flow Missing

Blue = yes, red = no


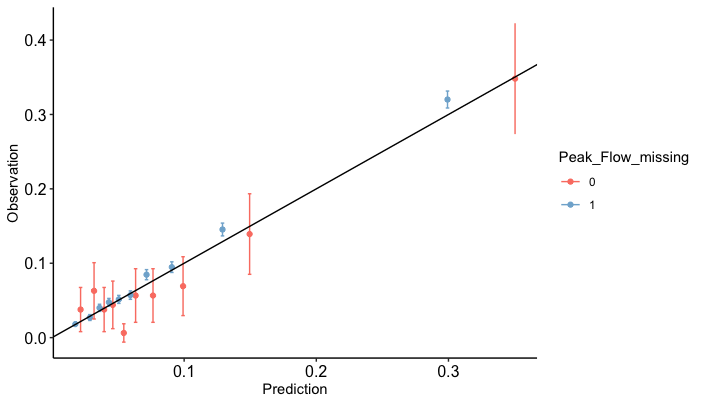


Blood Eosinophil Counts Missing

Blue = yes, red = no


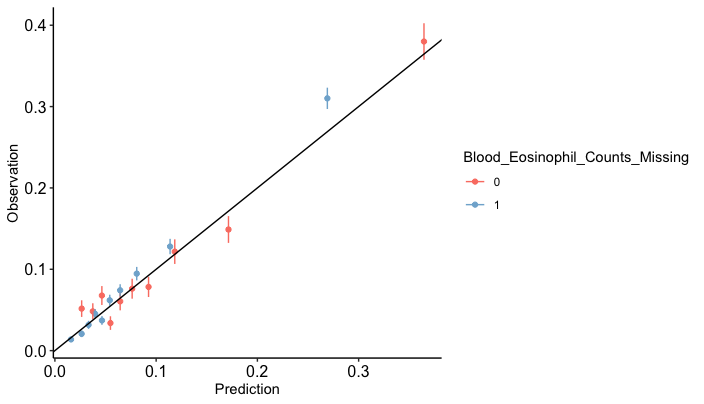


Recent respiratory infection

Blue = yes, red = no


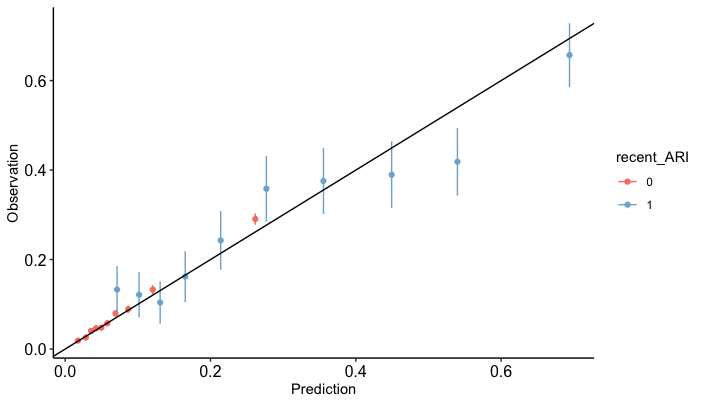


Recent asthma primary care encounter

Blue = yes, red = no


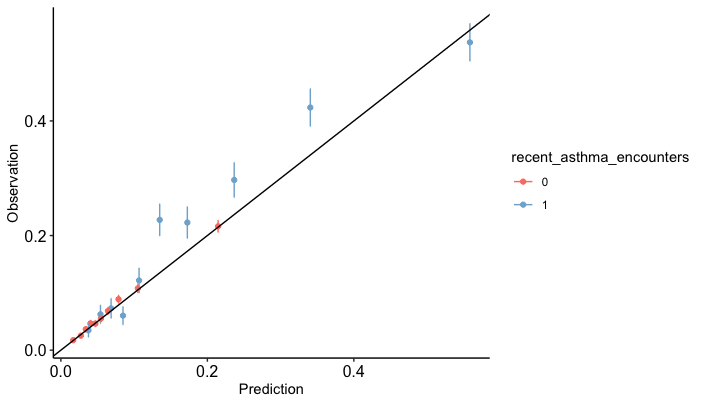


Recent steroid prescription in primary care

Blue = yes, red = no


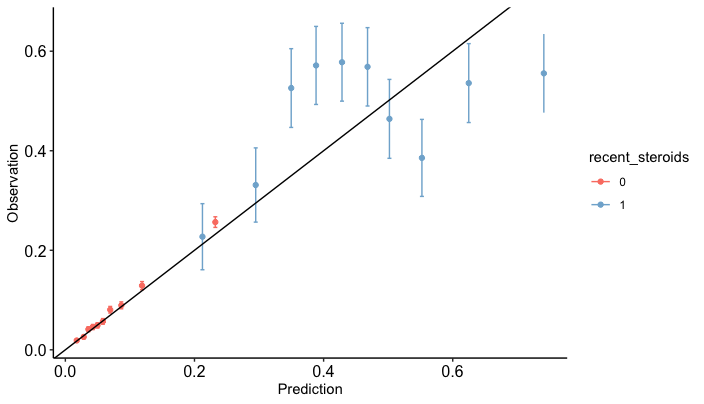


Never Smoked

Blue = yes, red = no


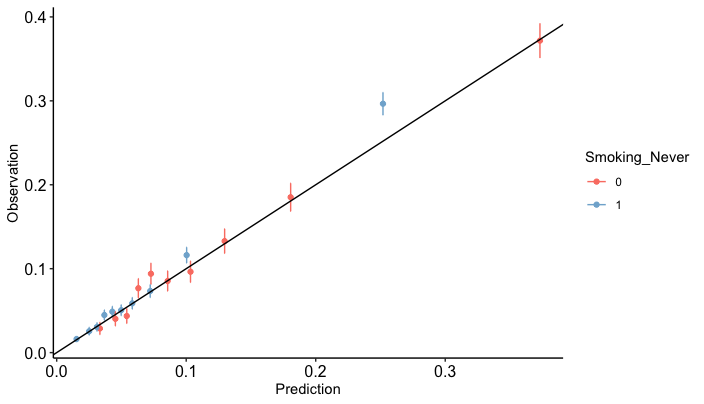


Asthma Attack Recorded in Primary Care in the last 2 years

Blue = None recorded, red = One or more recorded


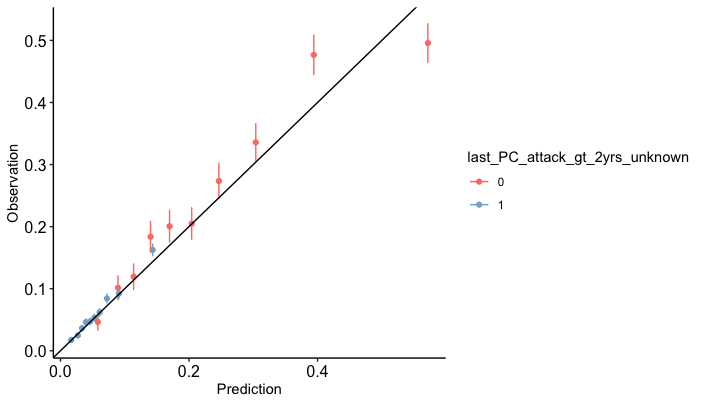


COPD Reference group

Blue = No diagnosis recorded, red = Diagnosis more than five years after asthma diagnosis, orange = diagnosed within five years of asthma diagnosis


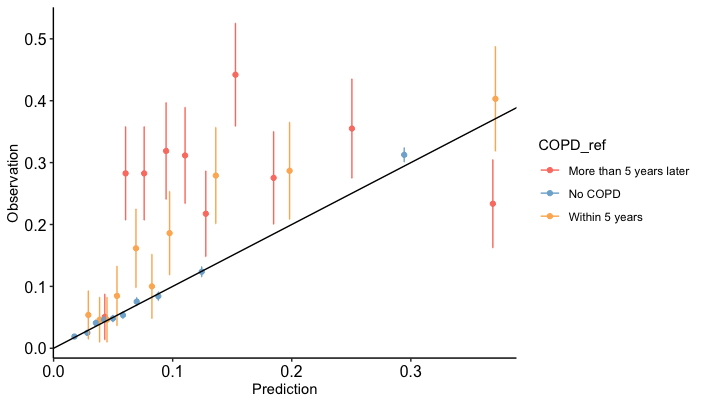


# Supplementary Materials References

1. British Thoracic Society, SIGN. *British Guideline on the Management of Asthma (2019 Edition)*.; 2019.

2. Scottish Government. *Review of Nomenclature of Units for Territorial Statistics (NUTS) Boundaries*.; 2016.

3. Scottish Government. *Scottish Government Urban Rural Classification 2016*.

4. Scottish Government National Statistics Publications. *Introducing The Scottish Index of Multiple Deprivation 2016*.; 2016.

5. Tibble H, Sheikh A, Tsanas A. Derivation of asthma severity from electronic prescription records using British thoracic society treatment steps. *BMC Pulmonary Medicine*. 2022;22(1):397. doi:10.1186/s12890-022-02189-3

6. Chawla N V., Bowyer KW, Hall LO, Kegelmeyer WP. SMOTE: Synthetic Minority Over-sampling Technique. *Journal of Artificial Intelligence Research*. 2002;16:321-357. doi:10.1613/jair.953

7. He H, Garcia EA. Learning from Imbalanced Data. *IEEE TRANSACTIONS ON KNOWLEDGE AND DATA ENGINEERING,*. 2009;21(9):1263-1284. doi:10.2174/156802608786786589

8. Torgo L. CRAN: Package “performanceEstimation” (version 1.1.0). *https://cran.r-project.org/web/packages/performanceEstimation/performanceEstimation.pdf*. Published online 2015.

9. Majka M. CRAN: Package ‘naivebayes’ (version 0.9.2). https://cran.r-project.org/web/packages/naivebayes/naivebayes.pdf.

10. Wright MN, Wager S, Probst P. CRAN: Package “ranger” (version 0.12.1). https://cran.r-project.org/web/packages/ranger/ranger.pdf.

11. Chen T, He T, Benesty M, et al. CRAN: Package “xgboost” (version 1.3.2.1). https://cran.r-project.org/web/packages/xgboost/xgboost.pdf.

12. Tibble H, Tsanas A, Horne E, et al. Predicting asthma attacks in primary care: protocol for developing a machine learning-based prediction model. *BMJ Open*. 2019;9(7):e028375. doi:10.1136/BMJOPEN-2018-028375

13. Price D, Wilson A, Chisholm A, et al. Predicting frequent asthma exacerbations using blood eosinophil count and other patient data routinely available in clinical practice. *Journal of Asthma and Allergy*. 2016;9:1. doi:10.2147/JAA.S97973

14. Blakey JD, Price DB, Pizzichini E, et al. Identifying Risk of Future Asthma Attacks Using UK Medical Record Data: A Respiratory Effectiveness Group Initiative. *Journal of Allergy and Clinical Immunology: In Practice*. 2017;5(4):1015-1024. doi:10.1016/j.jaip.2016.11.007

15. Kerr KF, Meisner A, Thiessen-Philbrook H, Coca SG, Parikh CR. RiGoR: Reporting guidelines to address common sources of bias in risk model development. *Biomarker Research*. 2015;3:2. doi:10.1186/s40364-014-0027-7

16. Collins GS, Reitsma JB, Altman DG, Moons KG. Transparent reporting of a multivariable prediction model for individual prognosis or diagnosis (TRIPOD): the TRIPOD Statement. *BMC Medicine*. 2015;13(1):1. doi:10.1186/s12916-014-0241-z

17. Benchimol EI, Smeeth L, Guttmann A, et al. The REporting of studies Conducted using Observational Routinely-collected health Data (RECORD) Statement. *PLOS Medicine*. 2015;12(10):e1001885. doi:10.1371/journal.pmed.1001885
