# Supplementary material for: Development and validation of a machine learning risk prediction model for asthma attacks in adults in primary care
Source: NPJ Prim Care Respir Med. 2025 Apr 23;35:24. doi: 10.1038/s41533-025-00428-8 (PMC12019439; doi:10.1038/s41533-025-00428-8)
Supplement: Supplementary file 2 — Reporting Guidelines Checklists [file 41533_2025_428_MOESM2_ESM.docx]

# Reporting Guidelines Checklists

Guidelines Used:

- RiGoR: Reporting Guidelines to address common sources of bias in Risk model development,
- TRIPOD: Transparent Reporting of a multivariable prediction model for Individual Prognosis Or Diagnosis,
- RECORD: Reporting of studies Conducted using Observational Routinely-collected health Data.

Note: RiGoR items related to participant recruitment (4a and 4b) and Biomarker Data (6 to 8 and 11) were not included as they were not relevant to this study.

| **Section/Topic** | **Checklist**  **(Item Number)** | **Checklist Item** | **Section** |
| --- | --- | --- | --- |
| Title and abstract | | | |
| Title | TRIPOD (1) | Identify the study as developing and/or validating a multivariable prediction model, the target population, and the outcome to be predicted. | Title |
|  | RECORD (1.1) | The type of data used should be specified in the title or abstract. When possible, the name of the databases used should be included. | Title |
|  | RiGoR (1) | Identify the article as reporting the development of a risk model combining multiple predictors (MeSH “Risk”, possibly “risk factor” and/or “biomarker”) | Title |
| Abstract | TRIPOD (2) | Provide a summary of objectives, study design, setting, participants, sample size, predictors, outcome, statistical analysis, results, and conclusions. | Abstract |
|  | RECORD (1.2) | If applicable, the geographic region and timeframe within which the study took place should be reported in the title or abstract. | Abstract |
|  | RECORD (1.3) | If linkage between databases was conducted for the study, this should be clearly stated in the title or abstract. | Abstract |
| Introduction | | | |
| Background and objectives | TRIPOD (3a) | Explain the medical context (including whether diagnostic or prognostic) and rationale for developing or validating the multivariable prediction model, including references to existing models. | Background |
|  | RiGoR (2) | Identify the overarching goal – why would an effective risk model be valuable to clinical care, public health, or research? | Background |
|  | TRIPOD (3b) | Specify the objectives, including whether the study describes the development or validation of the model or both. | Background |
| Methods | | | |
| Source of data | TRIPOD (4a) | Describe the study design or source of data (e.g., randomized trial, cohort, or registry data), separately for the development and validation data sets, if applicable. | Methods: Data |
|  | TRIPOD (4b) | Specify the key study dates, including start of accrual; end of accrual; and, if applicable, end of follow-up. | Methods: Data |
|  | RECORD (12.3) | State whether the study included person-level, institutional-level, or other data linkage across two or more databases. The methods of linkage and methods of linkage quality evaluation should be provided. | Supplementary Material A |
| Participants | TRIPOD (5a), RiGoR (3) | Specify key elements of the study setting (e.g., primary care, secondary care, general population) including number and location of centres. | Methods: Data |
|  | TRIPOD (5b), RECORD (6.1),  RiGoR (3) | Describe eligibility criteria for participants. The methods of study population selection (such as codes or algorithms used to identify subjects) should be listed in detail. If this is not possible, an explanation should be provided. | Methods: Analysis Population,  GitHub repository |
|  | RiGoR (5) | Describe the study design. | Background |
|  | RECORD (6.2) | Any validation studies of the codes or algorithms used to select the population should be referenced. If validation was conducted for this study and not published elsewhere, detailed methods and results should be provided. | Not Applicable |
|  | RECORD (6.3) | If the study involved linkage of databases, consider use of a flow diagram or other graphical display to demonstrate the data linkage process, including the number of individuals with linked data at each stage. | Figure 1 |
|  | TRIPOD (5c) | Give details of treatments received, if relevant. | Not Applicable |
| Outcome | TRIPOD (6a), RiGoR (9) | Clearly define the outcome that is predicted by the prediction model, including how and when assessed. | Methods: Outcome Ascertainment |
|  | TRIPOD (6b) | Report any actions to blind assessment of the outcome to be predicted. | Not Applicable |
|  | RECORD (7.1) | A complete list of codes and algorithms used to classify exposures, outcomes, confounders, and effect modifiers should be provided. If these cannot be reported, an explanation should be provided. | GitHub repository |
| Predictors | TRIPOD (7a), RiGoR (12a) | Clearly define all predictors used in developing or validating the multivariable prediction model, including how and when they were measured. | Supplementary Material B,  GitHub repository |
|  | TRIPOD (7b) | Report any actions to blind assessment of predictors for the outcome and other predictors. | Not Applicable |
|  | RiGoR (15) | For multi-center studies with the possibility of confounding by center, describe methods for adjusting or accounting for center effects. | Methods: Analysis Population |
| Sample size | TRIPOD (8) | Explain how the study size was arrived at. | Supplementary Material A,  Figure 4 |
| Missing data | TRIPOD (9), RiGoR (16) | Describe how missing data were handled (e.g., complete-case analysis, single imputation, multiple imputation) with details of any imputation method. | Methods: Analysis Population,  Supplementary Material B. |
| Statistical analysis methods | TRIPOD (10a) | Describe how predictors were handled in the analyses. | Supplementary Material B,  GitHub repository |
|  | TRIPOD (10b), RiGoR (12b) | Specify type of model, all model-building procedures (including any predictor selection), and method for internal validation. | Methods: Analysis Plan,  Supplementary Material C |
|  | RiGoR (13) | Document methodology used to develop risk model or classifier | Methods: Analysis Plan,  Supplementary Material C,  Supplementary Material G |
|  | RiGoR (12c) | Describe how model-selection bias was addressed in assessing the performance of final reported model(s). If model-selection bias was not addressed, state this explicitly. | Methods: Analysis Plan |
|  | TRIPOD (10d), RiGoR (10) | Specify all measures used to assess model performance and, if relevant, to compare multiple models. | Methods: Analysis Plan |
|  | RiGoR (14a) | Document methodology to avoid or correct for resubsitution bias in measures of the performance of the final reported model(s). | Methods: Analysis Plan |
|  | RiGoR (14b, 14c) | If an independent validation “test” dataset was used, document that the test data were not used for any part of model development, including variable selection. Document that these data were accessed only when models were finalized. Report the number of models evaluated on the “test” data and how these were selected. If cross-validation was used, state how final reported model was derived. | Methods: Analysis Plan |
|  | RiGoR (17) | Describe methods for assessing model calibration | Methods: Analysis Plan |
| Risk groups | TRIPOD (11) | Provide details on how risk groups were created, if done. | Methods: Analysis Plan |
| Data access and  cleaning methods | RECORD (12.1) | Authors should describe the extent to which the investigators had access to the database population used to create the study population. | Methods: Data Management, Ethics, and Reporting |
|  | RECORD (12.2) | Authors should provide information on the data cleaning methods used in the study. | Supplementary Material B,  GitHub repository |
|  | RECORD (22.1) | Authors should provide information on how to access any supplemental information such as the study protocol, raw data, or programming code. | Methods: Data Management, Ethics, and Reporting |
| Results | | | |
| Participants | TRIPOD (13a), RECORD (13.1) | Describe the flow of participants through the study, including the number of participants with and without the outcome and, if applicable, a summary of the follow-up time. A diagram may be helpful. | Results: Analysis Population,  SupplementaryMaterial A,  Figure 1,  Figure 2,  Figure 4 |
|  | TRIPOD (13b), RiGoR (18) | Describe the characteristics of the participants (basic demographics, clinical features, available predictors), including the number of participants with missing data for predictors and outcome. | Table 1 |
| Model development | TRIPOD (14a) | Specify the number of participants and outcome events in each analysis. | Results: Analysis Population |
|  | TRIPOD (14b) | If done, report the unadjusted association between each candidate predictor and outcome. | Not Applicable |
| Model specification | TRIPOD (15a), RiGoR (19) | Present the full prediction model to allow predictions for individuals (i.e., all regression coefficients, and model intercept or baseline survival at a given time point). | Not Applicable |
|  | TRIPOD (15b) | Explain how to the use the prediction model. | Not Applicable |
| Model performance | TRIPOD (16), RiGoR (20) | Report performance measures (with CIs) for the prediction model. | Not Applicable |
| Model Calibration | RiGoR (21) | Assess and report evidence of risk model calibration | Results: Discrimination and Calibration in Population Subgroups |
| Discussion | | | |
| Limitations | TRIPOD (18), RiGoR (23) | Discuss any limitations of the study (such as nonrepresentative sample, few events per predictor, missing data). | Discussion: Strengths and Limitations |
|  | RECORD (19.1) | Discuss the implications of using data that were not created or collected to answer the specific research question(s). Include discussion of misclassification bias, unmeasured confounding, missing data, and changing eligibility over time, as they pertain to the study being reported. | Discussion: Results in Context |
| Interpretation | TRIPOD (19b) | Give an overall interpretation of the results, considering objectives, limitations, and results from similar studies, and other relevant evidence. | Discussion: Results in Context |
| Implications | TRIPOD (20), RiGoR (22) | Discuss the potential clinical use of the model and implications for future research. | Discussion: Results in Context |
| Other information | | | |
| Supplementary information | TRIPOD (21) | Provide information about the availability of supplementary resources, such as study protocol, Web calculator, and data sets. | Methods: Analysis Plan,  Methods: Data Management, Ethics, and Reporting |
| Funding | TRIPOD (22) | Give the source of funding and the role of the funders for the present study. | Funding |
